# Harnessing Antioxidants for Abiotic Stress Management: Mechanistic Insights and Prospects for Sustainable Agriculture

**DOI:** 10.3390/antiox15030337

**Published:** 2026-03-07

**Authors:** Fasih Ullah Haider, Tianhao Liu, Luis Carlos Ramos Aguila, Babar Shahzad, Habiba, Peng Zhang, Xiangnan Li

**Affiliations:** 1 Key Laboratory of Black Soil Conservation and Utilization, Northeast Institute of Geography and Agroecology, Chinese Academy of Sciences, Changchun 130102, China; haider281@iga.ac.cn (F.U.H.); liutianhao@iga.ac.cn (T.L.); 2 State Key Laboratory of Biocontrol, School of Ecology, Sun Yat-sen University, Shenzhen 518000, China; carlos3@mail.sysu.edu.cn; 3 School of Agriculture, Food and Wine, Adelaide University, Adelaide, SA 5064, Australia; babar.shahzad@adelaide.edu.au; 4 Department of Biological Science, Science Hall-3401, Lehman College-CUNY, Bronx, NY 10468, USA; fnu.habiba79@login.cuny.edu

**Keywords:** abiotic stress, reactive oxygen species, redox signalling, ascorbate–glutathione cycle, peroxiredoxin–thioredoxin, NADPH, genome editing, crop resilience

## Abstract

Abiotic stresses disrupt redox homeostasis and reduce crop productivity. Antioxidant networks support resilience by limiting excess reactive oxygen species (ROS) and maintaining redox signalling for stress perception, gene expression, and metabolic reprogramming. We summarize advances (2000–2025) in ROS generation, detoxification mechanisms, and signalling across organelles, including chloroplasts, mitochondria, peroxisomes, and the apoplast. This includes compartmentalized enzymes—superoxide dismutase (SOD), catalase (CAT), ascorbate peroxidase (APX), glutathione peroxidase (GPX), and glutathione reductase (GR)—as well as the peroxiredoxin–thioredoxin system and non-enzymatic buffers like ascorbate, glutathione, tocopherols, carotenoids, and flavonoids. We uniquely synthesize these findings in a compartment-resolved “redox rheostat” model, linking ROS concentration–time windows (signaling vs. damage) to antioxidant network design (kinetic tiers, compartmentation, and trade-offs) and identifying intervention points for breeding, genome editing, and field-scale priming. We emphasize constraints, such as NADPH supply and antioxidant recycling capacity, that lead to context-dependent outcomes. We evaluate omics, transgenic strategies, genome editing (CRISPR and Cas systems), exogenous applications, and plant–microbe associations. This synthesis clarifies how antioxidant systems protect photosynthetic and respiratory machinery while supporting signalling, thus outlining routes to climate-resilient, yield-stable crops across varied environments and stresses.

## 1. Introduction

Agriculture is the cornerstone of global food security, yet it increasingly faces diverse climatic threats [1]. Rapid shifts in climate patterns, rising temperatures, altered precipitation, intensifying droughts, soil salinization, and recurrent heatwaves represent some of the toughest challenges to crop stability and productivity worldwide [2]. Quantitative data underscore these effects: abiotic stresses cause yield losses in major crops such as wheat (*Triticum aestivum* L.), rice (*Oryza sativa* L.), and maize (*Zea mays* L.), with magnitude varying by stress type, severity, timing, genotype, and environment [3,4]. Empirical models show that warming results in about 3–7% yield loss per 1 °C in major cereals, with disproportionate losses in vulnerable tropical and subtropical regions [5,6]. By mid-century, many projections indicate that combined climatic stresses could greatly reduce cereal productivity without adaptation, increasing pressure on global food security as populations grow [7]. Thus, it is essential to broaden our understanding of plant physiological and molecular responses to abiotic stress and boost crop resilience for sustainable agriculture [8,9,10].

A common feature among different abiotic stressors is their strong disruption of cellular redox homeostasis [1,2,3,4]. Under normal conditions, plants naturally produce reactive oxygen species (ROS). These act as metabolic switches within organelles such as chloroplasts, mitochondria, and peroxisomes [11]. ROS, which include superoxide radicals (O_2_^•−^), hydrogen peroxide (H_2_O_2_), hydroxyl radicals (−OH), and singlet oxygen (^1^O_2_), usually exist at low basal levels. They serve important roles as secondary messengers in processes like development, hormone signalling, stomatal regulation, and pathogen defence [12,13]. When abiotic stress occurs, this balance is disrupted by excess ROS production [8]. For example, drought stress can rapidly increase hydrogen peroxide levels in leaf mesophyll cells, whereas saline exposure triggers oxidative bursts primarily in roots [12]. Though ROS are critical for signalling, excessive amounts lead to oxidative damage. This affects membrane lipids, protein complexes (including those for photosynthesis), and nucleic acids [13]. Thus, ROS are both vital messengers and potential cytotoxins. Their regulation is crucial for plant survival under stress [14,15].

Plants fight oxidative damage through a complex antioxidant defence network that includes both enzymatic and non-enzymatic components. This network maintains redox balance and supports adaptive signalling [4,6,16]. Enzymatic antioxidants include superoxide dismutase (SOD), catalase (CAT), and ascorbate peroxidase (APX). These enzymes detoxify ROS efficiently and specifically [17]. For instance, SOD converts superoxide radicals into H_2_O_2_ at a very rapid rate. This allows enzymes like CAT to further break down H_2_O_2_ into harmless water and oxygen, preventing the formation of dangerous hydroxyl radicals [8,13]. Alongside these are non-enzymatic antioxidants. These include ascorbic acid (vitamin C), glutathione (GSH), carotenoids, tocopherols, and various phenolic and flavonoid compounds. They help buffer ROS levels and support redox-sensitive signaling pathways critical for stress acclimation [18,19,20]. Genetic studies show the importance of antioxidants. Rice plants that overexpress chloroplast-targeted SOD or APX show better stress tolerance. In contrast, GSH biosynthesis mutants are more susceptible to oxidative stress [21]. Thus, antioxidants are dynamic regulators that shape cellular signalling, stress memory, and flexibility during adaptation to abiotic stress [22].

Despite progress in recent decades, we still lack a full understanding of how antioxidant systems integrate with broader cellular stress networks. Crosstalk between ROS signalling and phytohormonal pathways, such as abscisic acid (ABA), salicylic acid (SA), jasmonates, and ethylene, is only partly understood, especially with respect to timing and feedback. Moreover, while applying antioxidants like APX or GSH externally can boost tolerance in controlled settings, their effectiveness in the field is unpredictable. Results vary across studies and depend on the form of application, including foliar, seed, and soil treatments [1,20]. Additionally, maintaining chronically high antioxidant activity has metabolic costs. This leads to trade-offs between stress tolerance and achieving optimal growth or yield [16,17]. Taken together, these challenges show the need for research that combines mechanistic detail with practical applications. Such approaches must link molecular biology to agronomy to realize the promise of antioxidants for crop improvement. In this review, we focus on causal evidence, genetic changes, targeted interventions, and yield validation, and note when claims rely mainly on short-term or inconsistent field results. Antioxidant-based strategies succeed only if they balance ROS signalling and prevent ongoing oxidative overload. This lesson should guide breeding and management decisions.

Abiotic stress tolerance depends on more than just antioxidant abundance. Rather, it is governed by the way these networks are organized across cell compartments. These networks act as a quantitative rheostat, keeping ROS within beneficial signalling ranges and preventing oxidative damage. This review addresses four main questions: (i) where and when ROS are produced under stress (Section 3); (ii) how compartmentalized antioxidant systems buffer redox signals (Section 4); (iii) how this regulation adapts under drought, salinity, temperature extremes, and heavy metals (Section 5); and (iv) which interventions can realistically adjust this rheostat in crops (Section 6 and Section 7), considering trade-offs, feasibility, and field constraints. Importantly, we assess antioxidant strategies in light of agronomic results, yield, stability, and quality, focusing on performance under combined or sequential stresses as the real test for field utility. By linking biological mechanisms to practical actions, our framework highlights recurring challenges, such as limited NADPH supply, insufficient recycling, and trade-offs between growth and defence. These issues explain why many antioxidant modifications do not translate to field success. Therefore, we discuss omics, transgenic, CRISPR, and field interventions only when they directly affect the ROS–antioxidant network and show real improvements in stress tolerance. Topics not central to redox regulation are not covered in detail.

## 2. Research Methodology

A structured literature search and evidence synthesis were conducted to examine antioxidant function in plants under abiotic stress. We searched databases (Web of Science, Scopus, PubMed, SpringerLink, ScienceDirect, and Google Scholar) for studies published from January 2000 to July 2025. Boolean combinations of terms covered abiotic stress, ROS/redox processes, antioxidant systems, and major stress classes (drought, salinity, temperature extremes, and heavy metals). We applied filters for translational approaches (transgenics, CRISPR/Cas, nanoparticles, and agronomic interventions). To address field relevance, we prioritized studies that reported agronomic outcomes (yield components, yield stability, or crop quality traits). We included evidence from combined or sequential stress designs (e.g., drought–heat, salinity–nutrient limitation) and multi-environment validation where available. Studies were screened for relevance and methodological quality, prioritizing recent advances (2015–2025) while retaining seminal work. The final synthesis is organized around four themes: ROS dynamics under stress; compartmentalized antioxidant network logic; stress-specific reparameterization; and intervention classes, including their feasibility, trade-offs, and field relevance.

## 3. Compartment-Specific ROS Dynamics Under Abiotic Stress (What Changes vs. Normal and Why It Matters)

**Figure 1:**
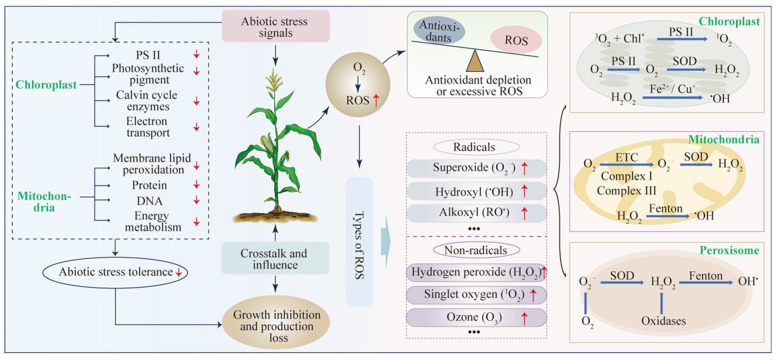
Reactive oxygen species (ROS) balance shifts from controlled signalling (normal) to harmful stress (abiotic stress). Under normal conditions, cells naturally produce ROS. Antioxidants keep these levels in check. This balance allows ROS to send helpful signals. Under abiotic stress, the balance breaks down. Antioxidants run low, and/or too much ROS builds up (red ↑). This disruption spreads as chloroplasts, mitochondria, and peroxisomes interact with one another. Chloroplasts (excited chlorophyll/PSII) make singlet oxygen (^1^O_2_) and superoxide radicals (O_2_^•−^). Superoxide dismutase (SOD) changes O_2_^•−^ into hydrogen peroxide (H_2_O_2_). Iron or copper helps make ^•^OH. Mitochondria mainly make O_2_^•−^ via their electron transport chains (ETCs). SOD then converts O_2_^•−^ to H_2_O_2_ and generates more ^•^OH. In peroxisomes, oxidases generate H_2_O_2_, leading to more ^•^OH. These changes between cell parts slow growth and lower yield. Stress-linked ROS are radicals (like O_2_^•−^, ^•^OH, RO^•^) and non-radicals (like H_2_O_2_, ^1^O_2_, O_3_); all rise under stress (red ↑). The red arrows indicate that stress reduces photosynthesis and respiration. This increases cell injury and lowers overall stress tolerance. Abbreviations: ROS, reactive oxygen species; SOD, superoxide dismutase; PSII, photosystem II; ETC, electron transport chain.

Abiotic stresses such as drought, salinity, temperature extremes, and heavy metal toxicity disrupt plant metabolism. They quickly disturb cellular redox homeostasis and change ROS production, accumulation, and signalling [1,3,7,23], [Figure 1]. Under normal conditions, ROS are produced continuously as by-products of aerobic metabolism. During stress, ROS can build up faster than the cell can detoxify them [24]. ROS serve as signalling messengers, but can also cause oxidative damage. How cells respond depends on the type of ROS, where they are produced, their amount, and how long they persist [20,24]. Understanding stress tolerance requires a detailed, compartment-specific view of ROS dynamics, rather than assuming more ROS is always harmful.

### 3.1. Sources of ROS in Plant Cells

Plant cells contain multiple ROS-generating sites that are interconnected through redox and Ca^2+^ signalling networks [6,7,8]. ROS produced in one compartment can influence dynamics in other compartments, illustrating an integrated cellular redox system rather than isolated organelle events [11,12,13].

#### 3.1.1. Chloroplasts: The Photosynthetic Hub of ROS

Chloroplasts are major sources of ROS under abiotic stress. Photosynthetic electron transport is highly sensitive to disruptions in CO_2_ assimilation and energy balance [25,26]. Drought, salinity, and heat often limit CO_2_ availability and carbon fixation. This leads to over-reduction in the electron transport chain and promotes O_2_^•−^ formation at PSI (Mehler-type reactions) [10,11,12], [Figure 1]. Stromal SOD rapidly converts O_2_^•−^ to H_2_O_2_. This links superoxide formation to downstream peroxide signalling and detoxification [27]. Excess excitation energy generates ^1^O_2_ at PSII. ^1^O_2_ acts locally due to its high reactivity and short lifetime [28]. H_2_O_2_ is more stable and diffusible. This enables chloroplast-to-nucleus retrograde signalling and transcriptional reprogramming during stress acclimation [29]. At physiological levels, chloroplast-derived ROS participate in photoprotective regulation (such as NPQ and antenna adjustments). Sustained ROS accumulation promotes photoinhibition, lipid peroxidation, and cell death [30]. Chloroplast ROS homeostasis provides a sensitive readout of the photosynthetic “redox budget” during stress.

#### 3.1.2. Mitochondria: Balancing Respiration and ROS

Mitochondria generate ROS mainly by electron leakage from complexes I and III of the respiratory electron transport chain during oxidative phosphorylation [Figure 1]. Under steady-state conditions, leakage is limited. Drought, chilling, and heavy metals slow downstream electron flow and increase electron transfer to O_2_, raising O_2_^•−^ formation [31]. Mn-SOD converts mitochondrial O_2_^•−^ to H_2_O_2_ in the matrix, and peroxidases detoxify it to support ROS homeostasis [32]. The alternative oxidase (AOX) pathway is a key adaptation. It relieves over-reduction in the ETC and reduces ROS generation, but decreases ATP yield. This supports survival under stress [33]. Mitochondrial H_2_O_2_ also functions in retrograde signalling and influences nuclear gene expression programmes. These programmes adjust antioxidant capacity and increase stress acclimation [34,35,36].

#### 3.1.3. Peroxisomes: Hotspots of Hydrogen Peroxide

Peroxisomes are prominent H_2_O_2_-producing organelles due to oxidative pathways. These include photorespiration (via glycolate oxidase), fatty acid β-oxidation, and purine catabolism [37], [Figure 1]. Photorespiration increases strongly under conditions that lower internal CO_2_, such as drought, high light, or salinity. This makes peroxisomes major contributors to cellular peroxide load and redox signalling [38]. H_2_O_2_ can diffuse and participate in inter-organelle communication. Thus, peroxisome-derived H_2_O_2_ can influence chloroplast and mitochondrial redox states and integrate stress signalling across compartments [39].

#### 3.1.4. Plasma Membrane NADPH Oxidases (RBOHs): Deliberate ROS Producers

Plasma membrane NADPH oxidases (RBOHs) generate ROS deliberately as part of signal transduction, unlike organelle-derived “leak” ROS [40]. RBOHs transfer electrons from cytosolic NADPH to apoplastic oxygen, producing O_2_^•−^, which is converted to H_2_O_2_ and then triggers downstream signalling. Their activity is tightly controlled by Ca^2+^ binding, phosphorylation, and interactions with regulatory proteins. This enables rapid, localized ROS production in response to stress cues [41]. For example, ABA-induced RBOH activation in guard cells promotes apoplastic H_2_O_2_ accumulation and Ca^2+^ influx. This contributes to stomatal closure [38]. In roots, RBOH-driven ROS bursts regulate ion transport and mediate stress-induced root architectural changes. They can also propagate as systemic ROS waves that coordinate whole-plant acclimation with Ca^2+^ signalling [30,31,32].

#### 3.1.5. Integration Across Organelles: A Redox Communication Network

ROS production is coordinated across compartments through amplification loops and signalling crosstalk. It is not confined to isolated sites [24]. Chloroplast ROS can influence mitochondrial ROS generation, a process called “ROS-induced ROS release.” This reinforces stress signalling. RBOH-mediated apoplastic ROS and Ca^2+^ waves support long-distance communication from local stress sites to distant tissues [38]. Peroxisomal H_2_O_2_ can connect photorespiratory flux with organellar redox status. This helps coordinate stress responses at the systems level [25]. The compartment of origin and the timing of ROS are decisive for cell fate. This balance enables plants to signal for acclimation while limiting oxidative damage.

### 3.2. ROS Homeostasis: The Fine Balance

ROS embody a central biological paradox: at controlled levels, they serve as indispensable messengers. When they exceed cellular buffering capacity, however, they become damaging oxidants [10,11,12]. This balance is determined by three interacting variables: ROS amplitude, subcellular distribution, and duration. Together, these determine whether ROS acts as a signal or a stressor [39]. Plants maintain ROS homeostasis via coordinated enzymatic and non-enzymatic antioxidants, redox-sensitive transcriptional regulation, and crosstalk with hormonal pathways [18,19,20]. Here, we use these control variables to formalize a compartment-resolved “redox rheostat.” This framework distinguishes signalling transients from damaging overload, providing a unifying logic for interpreting antioxidant responses across stresses.

#### 3.2.1. Beneficial Thresholds and the Signaling Role of ROS

At low-to-moderate levels (often in the low micromolar range for H_2_O_2_, ROS act as second messengers. Their biological specificity comes from their limited lifetime and spatial confinement [40]. In guard cells, for example, transient H_2_O_2_ elevations activate Ca^2+^-dependent kinase cascades. These cascades drive stomatal closure during drought responses [1,5,9,10]. In photosynthetic tissues, ^1^O_2_ generated at PSII is short-lived and locally restricted. Yet, it can initiate retrograde signalling pathways that induce nuclear protective programmes and acclimation [15,20]. Similarly, localized apoplastic H_2_O_2_ gradients contribute to root growth and cell-wall remodelling. These instances show that ROS convey spatial and temporal information, not functioning solely as metabolic by-products [23,25].

#### 3.2.2. Harmful Thresholds: The Switch to Oxidative Stress

When ROS accumulation becomes sustained and exceeds antioxidant capacity, the signalling regime collapses. For example, high cytosolic H_2_O_2_ in the tens to hundreds of micromolar range drives direct cellular injury [32,36]. The ^•^OH, produced via Fenton chemistry, is particularly destructive. It reacts with DNA, proteins, and membrane lipids at the site of formation, causing irreversible oxidative damage [15,20]. Downstream consequences include lipid peroxidation, accumulation of cytotoxic aldehydes (e.g., MDA), and impairment of protein function. If ROS remain elevated, they activate programmed cell death and accelerate senescence [23,34,41]. Thus, ROS intensity and persistence act as a cell-fate switch. Transient, buffered pulses support acclimation, while sustained overload promotes cytotoxicity and tissue decline [34].

#### 3.2.3. Crosstalk Between ROS and Hormonal Networks

ROS homeostasis is tightly integrated with hormone signalling. Hormones shape ROS production and help interpret ROS signals in a context-dependent manner [34,35,36]. ABA promotes RBOH-dependent apoplastic ROS generation in guard cells, amplifying Ca^2+^ signalling and enabling rapid stomatal closure. ROS can also enhance ABA sensitivity, reinforcing drought responses [15,38]. Salicylic acid increases ROS accumulation by modulating ROS production and suppressing detoxification pathways, such as CAT, thereby amplifying defence signalling and priming loops [8,23]. Jasmonates frequently balance these responses by inducing antioxidant enzymes that keep ROS within a signalling-competent window during wounding or salinity responses [42]. Ethylene links ROS dynamics to development and ageing. Transient ROS can support adaptive growth responses, while chronic ROS contributes to senescence [43]. Hormone–ROS coupling encodes both the amplitude and meaning of ROS signals.

#### 3.2.4. ROS as Systemic Signals: ROS Waves and Whole-Plant Communication

ROS signalling does not stay confined to local production sites. RBOH-driven ROS waves can propagate through tissues and along vasculature, often coupled to Ca^2+^ waves. This enables rapid long-distance communication of stress status across the plant [20,44]. Such systemic signalling lets unstressed organs pre-activate protective programmes, anticipating stress spreading through the plant [40]. Antioxidant systems do not eliminate these waves. Instead, they shape signal amplitude and prevent escalation into uncontrolled oxidative damage, preserving communication while keeping redox safety [25].

Overall, ROS behaviour under abiotic stress is a balance between adaptive signalling and oxidative injury. This balance is quantitative and compartment-dependent. When ROS are produced as transient, spatially restricted pulses within buffering capacity, they coordinate gene expression, metabolic adjustments, and developmental acclimation. But when ROS production becomes sustained or widespread, they drive lipid peroxidation, protein dysfunction, and cell death. The main determinants of this equilibrium are organelle electron-transport kinetics, antioxidant recycling capacity, hormone–ROS integration, and systemic signalling. These factors shape whether plants acclimate or succumb under stress. They represent central leverage points for developing climate-resilient crops.

## 4. Antioxidant Systems in Plants

### 4.1. Enzymatic Antioxidants

**Figure 2:**
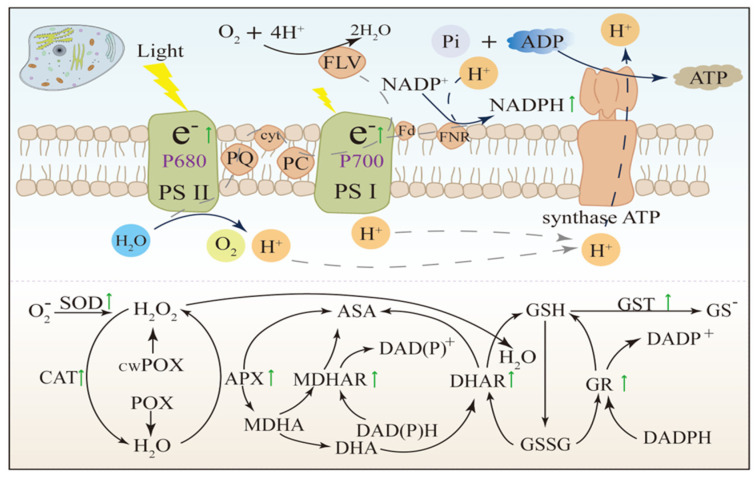
Chloroplast electron transport links stress-induced ROS (reactive oxygen species) to detoxification networks that maintain redox balance. In non-stressed chloroplasts, light-driven electron flow moves from PSII (P680) to PSI (P700) through PQ, cyt, and PC. This process helps form NADPH via FNR and generates a proton gradient for ATP synthesis. During environmental stress, excess electrons leak to O_2_, increasing ROS like O_2_^•−^ and H_2_O_2_, which can damage the photosystems if not controlled. Chloroplasts manage ROS through antioxidant systems. SOD converts O_2_^•−^ to H_2_O_2_, which is then broken down by CAT, peroxidases (POX; cwPOX), or the ascorbate–glutathione cycle (APX, MDHAR, DHAR, GR). GST helps buffer redox changes and aids detoxification. Green ↑ indicates increased activity of protective antioxidant systems. Abbreviations: PSII/PSI, photosystems II/I; PQ, plastoquinone; cyt, cytochrome complex; PC, plastocyanin; Fd, ferredoxin; FNR, ferredoxin–NADP^+^ reductase; FLV, flavodiiron proteins; APX, ascorbate peroxidase; MDHAR, monodehydroascorbate reductase; DHAR, dehydroascorbate reductase; GR, glutathione reductase; GST, glutathione S-transferase; ASA, ascorbate; GSH/GSSG, reduced/oxidized glutathione.

Plants rely on a sophisticated enzymatic defence system to maintain ROS homeostasis, with each enzyme playing a distinct yet interconnected role [1,15,20]. By detoxifying ROS and integrating into redox signalling pathways, these enzymes ensure that ROS are channelled into adaptive functions rather than unchecked damage [23,32,38], [Figure 2]. This interconnectedness lays the groundwork for understanding how individual enzymes operate within the broader network.

#### 4.1.1. Superoxide Dismutase (SOD)

Superoxide dismutase (SOD) is the primary enzymatic barrier against O_2_^•−^. It serves as the first defence against uncontrolled radical accumulation under stress [44]. O_2_^•−^ is generated mainly by photosynthetic and respiratory electron transport. This radical is highly reactive and can damage Fe–S clusters in metabolic enzymes, disrupt carbon fixation, and promote the formation of more toxic ROS through redox cycling [5,23,45]. SOD rapidly converts O_2_^•−^ into H_2_O_2_ and molecular oxygen according to 2O_2_^•−^ + 2H^+^ → H_2_O_2_ + O_2_. It operates at near diffusion-limited rates (~109 M^−1^ s^−1^) [44]. This reaction is protective, stopping local superoxide damage and forming H_2_O_2_, which is more stable and can be detoxified or used in signalling [46,47]. Plants express several SOD isoforms, placing them in key ROS-producing sites for “interception at the source.” Cu/Zn-SOD is usually in the cytosol and chloroplasts [Figure 2]. It buffers O_2_^•−^ produced during photosynthetic stress and helps maintain photosystem performance and carbon assimilation steady when CO_2_ fixation declines [46]. Mn-SOD is found in the mitochondrial matrix. It protects respiratory complexes during stresses that increase electron leakage, such as drought, chilling, or heavy metal exposure [10]. Fe-SOD, present in the chloroplasts of species such as maize and *Arabidopsis*, maintains chloroplast redox balance during stress that involves photorespiratory and photochemical ROS [23]. Compartment-specific deployment is essential because O_2_^•−^ is short-lived and poorly diffusible. Effective scavenging must occur near its generation site [45]. Experimental evidence shows SOD is quantitatively important for stress tolerance. Salinity stress strongly induces mitochondrial Mn-SOD activity, which coincides with reduced O_2_^•−^ accumulation and improved respiratory performance [44]. Transgenic tobacco (*Nicotiana tabacum* L.) lines overexpressing chloroplast Cu/Zn-SOD show better photosynthesis and less photoinhibition under high light [25]. *Arabidopsis* mutants lacking Fe-SOD have more lipid peroxidation under drought, highlighting this isoform’s role in protecting membranes and photosynthetic integrity during stress [22,37]. SOD also shapes redox signalling. It channels O_2_^•−^ into H_2_O_2_, allowing downstream processing by catalase, peroxidases, and the ascorbate–glutathione cycle. This enzyme also supports organelle-to-nucleus communication, which is needed for acclimation [46,47,48]. So, SOD acts not only as a detoxification enzyme but also as a gatekeeper. It turns a reactive radical into a regulated peroxide signal in compartment-specific antioxidant networks.

#### 4.1.2. Catalase (CAT)

Catalase is a major enzyme that removes H_2_O_2_ and maintains redox equilibrium under basal and stress conditions [44]. CAT catalyzes the breakdown of H_2_O_2_ to water and oxygen (2H_2_O_2_ → 2H_2_O + O_2_) without using reductants, making it an efficient route for peroxide detoxification [49]. Predominantly found in peroxisomes, CAT manages the high H_2_O_2_ flux produced during photorespiration and oxidase-dependent pathways [23]. Without rapid CAT activity, peroxisomal peroxide can exceed the capacity of lower-capacity peroxidases, increasing the risk of oxidative injury [38]. CAT’s high turnover rate (kcat ~10^7^ s^−1^) makes it effective for detoxification, though its substrate affinity is low (Km in the millimolar range) [50]. Thus, CAT acts as a high-capacity, low-affinity “safety valve” during peroxide surges. APX and peroxiredoxins are more efficient at lower (micromolar) H_2_O_2_ levels needed for signalling [51]. These combined properties establish a hierarchy: peroxidases fine-tune H_2_O_2_ levels at the cellular level, while CAT responds to sharp increases in H_2_O_2_ during stress. CAT activity often increases under abiotic stress, supporting oxidative protection. For example, drought-stressed maize shows a substantial rise in CAT activity, reflecting greater H_2_O_2_ detoxification [52]. In salt-stressed wheat, CAT induction correlates with reduced lipid peroxidation and better chlorophyll retention, supporting photosynthesis [46]. Besides detoxification, hormonal regulation influences CAT’s role in redox signalling. Salicylic acid (SA) can transiently inhibit CAT, allowing localized H_2_O_2_ buildup to initiate defence gene expression and priming [23,48,53]. Mechanistically, CAT acts as both a peroxide scavenger and a signalling gatekeeper: it prevents harmful H_2_O_2_ overload but permits controlled, localized H_2_O_2_ signals as needed [49,50,51,54]. This dual function places CAT at the centre of peroxide homeostasis and stress adaptation in plants.

#### 4.1.3. Ascorbate Peroxidase (APX)

Ascorbate peroxidase (APX) is a high-affinity peroxide scavenger. It fine-tunes H_2_O_2_ levels at low, signalling-relevant concentrations. In this role, APX complements CAT, which is optimized for bulk peroxide removal [5,15,34]. Specifically, APX reduces H_2_O_2_ to water (H_2_O_2_ + Asc → 2H_2_O + DHA) using ascorbate (Asc) as an electron donor. This enzyme operates efficiently in the micromolar range due to its relatively high substrate affinity (K_m_ ≈ 20–50 μM for H_2_O_2_) [55]. Thanks to this kinetic property, APX can remove excess H_2_O_2_ without abolishing ROS-dependent signalling. Consequently, APX helps maintain redox homeostasis during stress acclimation [27,53]. Notably, APX comprises multiple isoforms tailored to distinct cellular compartments, which aligns with the need for localized peroxide control [56]. For instance, cytosolic APX buffers H_2_O_2_ near signalling hubs and membranes. Similarly, chloroplast isoforms (stromal and thylakoid-bound APX) protect the photosynthetic apparatus from light-driven oxidative bursts [57]. In addition, peroxisomal APX complements catalase by processing micromolar levels of H_2_O_2_, while CAT handles higher-flux peroxide surges [58]. Mitochondrial APX also contributes by protecting respiratory function, detoxifying peroxide during oxidative phosphorylation, especially when electron flow is perturbed by stress [27]. Supporting the functional importance of APX, genetic and physiological studies have shown that Arabidopsis mutants lacking thylakoid APX exhibit increased lipid peroxidation and impaired PSII performance under high light. This evidence highlights APX’s role in photoprotection [43]. Conversely, APX overexpression in crop systems has been associated with reduced H_2_O_2_ accumulation, improved membrane integrity, and enhanced stress survival under salinity or drought [21,59]. Mechanistically, APX operates within the ascorbate–glutathione (AsA–GSH) cycle. The DHA produced by APX is recycled back to ascorbate by DHAR, using reduced glutathione (GSH) as a cofactor. This process links peroxide detoxification to cellular redox buffering and NADPH-dependent recycling [23,59]. Through this coupling, APX acts as both a detoxification enzyme and a signal-preserving modulator, maintaining H_2_O_2_ within a physiological window that supports acclimation while preventing escalation into oxidative damage [54,56,57].

#### 4.1.4. Glutathione Peroxidase (GPX)

Glutathione peroxidases (GPXs) detoxify H_2_O_2_ and, importantly, organic hydroperoxides (ROOH). They play a key role in limiting lipid hydroperoxides that accumulate in membranes under stress [44]. Plant GPXs are usually selenium-independent cysteine-based enzymes. This differs from many animal selenoprotein GPXs. They are distributed across chloroplasts, mitochondria, and the cytosol. These are sites where ROS generation intersects with peroxidation-prone lipids [55,60,61]. GPX uses reduced glutathione as an electron donor to reduce ROOH to the corresponding alcohol (ROH) (ROOH + 2GSH → ROH + GSSG + H_2_O). This interrupts lipid peroxidation chain reactions. Without this, the integrity of the thylakoid and mitochondrial membranes could be compromised, impairing photosynthesis and respiration [55,61]. Stress-responsive induction of GPX supports a protective role for membrane function. Basal activities are typically measurable in healthy tissues and often increase under drought, salinity, or heavy-metal exposure. This is consistent with inducible antioxidant defence [62,63]. In transgenic rice, GPX overexpression has been associated with reduced lipid peroxidation (lower MDA), improved membrane stability, and better maintenance of chlorophyll under salinity stress [64]. Similarly, increased GPX activity in *Arabidopsis* has been linked to reduced electrolyte leakage under cadmium (Cd) stress. This is consistent with improved membrane protection [65]. Functionally, GPX activity is integrated with glutathione recycling. The GSSG formed during peroxide reduction is converted back to GSH by glutathione reductase (GR) in an NADPH-dependent reaction. This couples the GPX function to the broader cellular redox economy and sustains turnover during stress [65,66]. Within the antioxidant hierarchy, GPXs complement APX and CAT by targeting peroxide species that directly threaten membranes, especially lipid hydroperoxides. They help preserve photosynthetic efficiency, mitochondrial energy metabolism, and overall cellular viability under abiotic stress [60,61]. This membrane-focused specialization makes GPX a critical component of oxidative stress tolerance, especially where lipid peroxidation is a dominant damage pathway.

#### 4.1.5. Glutathione Reductase (GR)

Glutathione reductase (GR) is a central enzyme in redox homeostasis. It regenerates reduced glutathione (GSH) from oxidized glutathione (GSSG) using NADPH (GSSG + NADPH → 2GSH + NADP^+^) [66,67]. This reaction sustains the AsA–GSH cycle. It enables continuous peroxide detoxification via APX, GPX, and associated recycling enzymes such as DHAR [68]. Without efficient GR-mediated recycling, GSH becomes depleted. GSSG accumulates, leading to a collapse of peroxide-scavenging capacity. The intracellular redox environment destabilizes under stress [69]. GR is present in multiple compartments. These include chloroplasts, mitochondria, cytosol, and peroxisomes. This distribution aligns with the widespread production of ROS and the demand for antioxidants. In chloroplasts, GR supports ascorbate turnover. It protects photosystems under high light and photooxidative stress [70]. Mitochondrial GR helps buffer ROS generated when electron transport is constrained. It preserves respiratory efficiency under adverse conditions [44]. In peroxisomes, GR aids detoxification of photorespiration-derived H_2_O_2_. It works with peroxidase systems [23]. GR’s stress responsiveness further highlights its role. GR activity often increases under abiotic stress. This maintains GSH pools and a high GSH/GSSG ratio. This ratio strongly associates with reduced lipid peroxidation and improved photosynthetic performance in stressed plants [15,71,72]. Mechanistically, GR links antioxidant function to cellular energy metabolism. It does this by coupling glutathione recycling to NADPH availability from photosynthetic reactions, the oxidative pentose phosphate pathway, and mitochondrial metabolism [73]. GR does not directly remove ROS. Instead, it acts as the redox “recycler.” GR determines the throughput and resilience of glutathione-dependent detoxification pathways during prolonged stress exposure [74].

#### 4.1.6. Peroxiredoxins and Thioredoxins

Peroxiredoxins (Prxs) and thioredoxins (Trxs) form an interconnected thiol-based redox system. This system combines peroxide detoxification with redox regulation and signalling [75]. Prxs are cysteine-dependent peroxidases that reduce H_2_O_2_ and organic hydroperoxides. In some contexts, they also reduce reactive nitrogen species. This reduction occurs via a conserved catalytic cysteine, which is oxidized during peroxide reduction and then restored by Trx via disulfide reduction [75,76]. Prxs typically exhibit high affinity for H_2_O_2_, often in the low micromolar range. They are well-suited to buffer signalling-range peroxide concentrations below the CAT efficient operating range. This supports fine control of basal redox status across compartments [77]. Prxs are widely distributed in chloroplasts, mitochondria, peroxisomes, and the cytosol, enabling localized regulation of ROS production and interpretation [5,38]. Beyond detoxification, Prxs can function as redox sensors and stress-responsive switches. Under stronger oxidative conditions, Prxs can become overoxidized at the catalytic cysteine. This transiently reduces peroxidase activity and, in some cases, promotes chaperone-like functions that support proteostasis during stress [78]. Trxs complement these roles as broad protein disulfide reductases. They relay reducing equivalents from NADPH (via thioredoxin reductase) to diverse targets [79]. In chloroplasts, Trx activity is also linked to light-driven electron flow through the ferredoxin–thioredoxin system. This couple’s photosynthetic energy status regulates the Calvin–Benson cycle enzymes and redox-sensitive metabolic steps [75]. This integration helps synchronize carbon metabolism, antioxidant capacity, and stress acclimation [23,34]. Genetic and physiological evidence support the importance of the Prx–Trx module in stress tolerance and photosynthetic stability. Perturbation of chloroplast Prx systems can increase peroxide accumulation and sensitize plants to high-light stress and photoinhibition. Enhanced Trx capacity has been associated with improved maintenance of photosynthesis and stress resilience in multiple systems [76,77,78,80]. Collectively, Prxs act as peroxide buffers and sensors. Trxs translate redox changes into reversible protein regulation, connecting ROS control to metabolic reprogramming during stress [81].

Collectively, enzymatic antioxidants function as a hierarchical, coupled network. SOD converts O_2_^•−^ to H_2_O_2_. Catalase removes high-flux peroxide. APX and GPX, together with the AsA–GSH cycle, regulate micromolar H_2_O_2_ and hydroperoxides. This regulation is relevant to signalling and membrane protection. GR sustains glutathione-dependent turnover through NADPH-driven recycling. The Prx–Trx module links peroxide buffering to redox regulation of metabolism and stress signalling. Through these interlinked tiers, plants maintain ROS within adaptive bounds. This preserves signalling while preventing oxidative damage across diverse abiotic stress conditions.

### 4.2. Non-Enzymatic Antioxidants

While enzymatic antioxidants provide highly regulated, compartmentalized ROS detoxification, plants also rely on diverse non-enzymatic antioxidants. These act as rapid scavengers, redox buffers, and essential cofactors [1,20]. Their metabolites help prevent ROS from exceeding damaging thresholds while preserving signalling functions. They integrate tightly with enzymatic pathways such as APX/GPX systems and thiol-based redox circuits, forming overlapping layers of redox control [23,34], [Figure 2].

#### 4.2.1. Ascorbic Acid (Vitamin C)

Ascorbic acid (Asc) is the dominant water-soluble antioxidant in plant cells. It serves as a central hub of redox metabolism [44]. Asc often accumulates in chloroplasts to high millimolar levels, typically 1–20 mM, and to higher concentrations in stress-acclimated tissues. This supports both direct ROS scavenging and turnover of the antioxidant network [81]. Asc directly quenches multiple ROS species, including highly reactive radicals. It is the preferred electron donor for ascorbate peroxidases (APXs) in H_2_O_2_ reduction, helping link non-enzymatic buffering to enzymatic peroxide control [82]. Beyond detoxification, Asc is a cofactor for violaxanthin de-epoxidase in the xanthophyll cycle. It supports non-photochemical quenching (NPQ) and photoprotection under excess light [83,84]. The function of Asc depends on pool size and rapid recycling. After donating electrons, Asc forms monodehydroascorbate (MDHA). MDHA can be reduced back to Asc by MDHAR using NAD(P)H or can disproportionate to dehydroascorbate (DHA) [82,85]. DHA is then reduced to Asc by DHAR in a GSH-dependent reaction. This integrates Asc turnover into the broader AsA–GSH cycle, linking Asc status to glutathione pools, GR activity, and NADPH supply [86]. This recycling capacity enables sustained APX turnover and stabilizes redox homeostasis across compartments, including chloroplasts, cytosol, mitochondria, and the apoplast [87]. Physiological and genetic evidence show Asc’s importance for stress resilience and photosynthetic stability. Asc-deficient mutants exhibit increased lipid peroxidation and accelerated photoinhibition under salinity or high light, indicating compromised ROS buffering and impaired NPQ [88,89]. Enhancing Asc recycling, for example, by DHAR overexpression, can sustain larger Asc pools and improve tolerance to drought and salinity. This supports the idea that recycling throughput is a key determinant of stress performance, not just Asc abundance [82]. In summary, Asc is both a direct antioxidant and an enabling cofactor. It coordinates photoprotection, peroxide detoxification, and redox signalling in response to abiotic stress.

#### 4.2.2. Glutathione (GSH)

Glutathione (GSH; γ-glutamyl-cysteinyl-glycine) is the most abundant non-protein thiol in plants and acts as a central “redox currency.” It supports detoxification, redox buffering, and signalling [90,91]. GSH typically occurs at millimolar concentrations in most tissues. Levels can rise further in chloroplasts, especially during stress acclimation [90]. Functionally, GSH controls ROS through several coupled pathways. It donates electrons for GPXs and glutathione S-transferases (GSTs), which reduce H_2_O_2_ and ROOH and limit lipid peroxidation in membranes [90,91,92,93,94]. GSH is also essential for the AsA–GSH cycle. In this cycle, DHAR uses GSH to regenerate Asc from DHA. This process sustains APX-dependent H_2_O_2_ detoxification and keeps peroxide levels within ranges compatible with signalling [95,96]. The ratio of reduced to oxidized glutathione (GSH/GSSG) sensitively reflects cellular redox status. This ratio often shifts under stress, indicating oxidative pressure and triggering antioxidant gene expression [94,96]. Stress often increases total GSH and induces GR, which reduces GSSG back to GSH using NADPH. These responses are frequently associated with improved redox buffering and reduced oxidative damage [97]. Genetic studies highlight GSH’s centrality. Mutants with impaired GSH biosynthesis show heightened sensitivity to oxidative and metal stress. Elevated GSH biosynthetic capacity commonly improves tolerance to salinity, ozone, and heavy metals [98,99,100]. Beyond buffering, GSH also participates in redox signalling through reversible S-glutathionylation. This process modulates protein activity and stability during stress responses [94,101]. In summary, GSH integrates detoxification, NADPH-dependent recycling, and signalling regulation, making it a core determinant of redox resilience under abiotic stress.

#### 4.2.3. Tocopherols (Vitamin E)

Tocopherols are lipophilic antioxidants enriched in plant membranes. The most abundant and biologically active isoform in many tissues is α-tocopherol [102]. Tocopherols are especially concentrated in chloroplast thylakoid membranes. In these locations, they protect polyunsaturated fatty acids from lipid peroxidation initiated by ROS (notably ^1^O_2_) and lipid peroxyl radicals formed during oxidative stress [102,103,104]. Tocopherols terminate peroxidation chain reactions by donating a hydrogen atom to lipid radicals. This produces a relatively stable tocopheroxyl radical, which can be recycled back to α-tocopherol by ascorbate and other reductants. This process functionally links tocopherol defence to the broader cellular antioxidant network [105,106]. Stress-responsive induction and mutant phenotypes demonstrate the importance of tocopherols for membrane stability and photoprotection. In *Arabidopsis*, α-tocopherol levels increase during drought, high light, and chilling, supporting its protective role during oxidative stress [107]. In contrast, tocopherol-deficient mutants (e.g., *vte1*) show pronounced lipid peroxidation, chlorosis, and accelerated senescence under high-light stress. This demonstrates that tocopherols are needed to restrain membrane oxidative cascades under high excitation pressure [105,108]. Tocopherols limit lipid peroxide amplification and stabilize membrane properties, preserving photosystem function and sustaining photosynthetic performance under abiotic stress [104,107,109]. Thus, tocopherols act as specialized membrane-phase antioxidants. They complement soluble redox buffers by protecting thylakoid integrity during stress.

#### 4.2.4. Carotenoids

Carotenoids are isoprenoid pigments serving two critical roles: as light-harvesting cofactors and as chloroplast antioxidants, making them central to photoprotection under abiotic stress [110]. By quenching triplet chlorophyll, they prevent ROS formation, and they also dissipate excess excitation energy that would otherwise produce ^1^O_2_. When oxidative load rises, carotenoids can chemically react with ROS [110,111,112]. β-carotene, located near reaction centres, is particularly important for suppressing ^1^O_2_ formation; it intercepts excited chlorophyll states, thereby protecting photosystem cores from oxidative damage [113,114]. In addition, carotenoids drive the xanthophyll cycle, a system that regulates non-photochemical quenching (NPQ) through rapid interconversion of violaxanthin, antheraxanthin, and zeaxanthin under fluctuating light and stress [115,116]. Accumulation of zeaxanthin is strongly associated with increased NPQ and reduced photoinhibition during high light or drought-related excitation pressure [117,118]. Genetic evidence further supports these functions. For example, mutants impaired in zeaxanthin formation (such as *Arabidopsis npq1*, deficient in violaxanthin de-epoxidase) exhibit enhanced photoinhibition and oxidative damage under high-light stress, which demonstrates the necessity of carotenoid-dependent energy dissipation for redox stability [119,120]. If oxidative stress exceeds buffering capacity, carotenoid oxidation can produce apocarotenoids, which act as signalling molecules linking chloroplast redox state to downstream acclimation programmes [104,121]. In sum, carotenoids coordinate ROS prevention, energy dissipation, and stress signalling, thus sustaining photosynthetic efficiency and chloroplast integrity under abiotic stress.

#### 4.2.5. Flavonoids, Phenolics, and Secondary Metabolites

Flavonoids and phenolic compounds comprise a diverse class of secondary metabolites that contribute to stress tolerance by combining direct antioxidant activity with photoprotection and modulation of signalling [5,15]. These compounds accumulate primarily in vacuoles, epidermal tissues, and the apoplast, positioning them at cellular interfaces where ROS exposure is high (e.g., under UV, drought, salinity, and oxidative bursts) [34]. Their polyhydroxylated structures enable them to donate electrons or hydrogen atoms to reactive species such as O_2_^•−^ and ^•^OH, forming resonance-stabilized radicals and thereby limiting oxidative chain reactions, particularly when enzymatic systems become saturated [122,123]. A significant additional function is UV screening and photoprotection. Flavonols such as quercetin and kaempferol absorb UV-B and reduce photooxidative excitation in underlying tissues, indirectly lowering chloroplast ROS formation and helping preserve photosynthetic capacity under high irradiance [124,125]. Phenolic acids (e.g., ferulic and caffeic acids) can further contribute by reinforcing cell walls through cross-linking and lignification-related chemistry, affecting both oxidative diffusion dynamics and stress resilience at the tissue level [123]. Genetic and physiological evidence support their protective role: mutants impaired in flavonoid biosynthesis (e.g., *Arabidopsis tt4*) exhibit elevated ROS levels and reduced tolerance to drought and UV stress, consistent with a loss of this peripheral antioxidant buffer [126]. Beyond scavenging, flavonoids can modulate hormone-linked growth programmes, for example, by affecting auxin transport, thereby integrating redox status with developmental adjustments under stress [122,127]. Thus, flavonoids and phenolics function as inducible, spatially targeted redox buffers that extend antioxidant protection beyond core organelles and couple oxidative cues to adaptive growth and defence responses.

### 4.3. Compartment-Specific Antioxidant Networks

ROS are generated in most plant compartments, but their effects depend on local production rates, diffusion, and strength of compartment-specific antioxidants [128]. To balance ROS’s dual role as toxic and signalling molecules, plants use spatially organized antioxidants tailored to each organelle’s metabolic needs [129]. Chloroplasts are especially vulnerable because photosynthesis produces O_2_^•−^ and ^1^O_2_ at PSI/PSII, particularly under excess light or CO_2_ shortage [130]. To cope, chloroplastic defences are both redundant and layered: SOD isoforms quickly convert O_2_^•−^ to H_2_O_2_. Stromal and thylakoid APXs keep H_2_O_2_ at signalling-friendly micromolar levels [131]. High ascorbate and glutathione pools support the AsA–GSH cycle and constant recycling via GR [15]. Membrane-phase antioxidants like tocopherols and carotenoids reduce lipid peroxidation and quench ^1^O_2_ in thylakoids [34]. Genetic data show this compartmental need: *Arabidopsis tapx* mutants have higher lipid peroxidation under high light, while tocopherol-deficient vte1 lines display chlorosis and faster senescence, highlighting the vital role of chloroplast antioxidants in photoprotection and photosynthetic stability [61,71].

Mitochondria generate ROS mainly at complexes I and III of the ETC. ROS leakage rises when electron flow is limited by drought, chilling, or heavy metals [88]. Mitochondrial Mn-SOD limits O_2_^•−^ buildup, while APX/GPX and thiol systems detoxify H_2_O_2_ and regulate redox-sensitive metabolism needed for respiration [35,132]. Stress-induced increases in these defences often lower oxidative damage and improve stress tolerance, supporting mitochondria’s central role in linking redox balance to energy metabolism [81]. Peroxisomes also act as high-flux peroxide organelles, making large amounts of H_2_O_2_ during photorespiration and other oxidase reactions [25]. Their antioxidant setup focuses on capacity: catalase rapidly clears H_2_O_2_ surges, while peroxisomal APX and Prxs fine-tune lower-level peroxide signals and support redox communication between organelles [132]. Peroxisomal antioxidant activity often increases under stresses that increase photorespiration, suggesting an adjustment to peroxide load [39].

The cytosol acts as an integration hub. It buffers ROS from organelles and maintains redox balance using soluble antioxidants and enzymes such as Cu/Zn-SOD, APX, and large ascorbate/glutathione pools [39,97]. Cytosolic scavenging shapes signalling precision by controlling H_2_O_2_ influx from the apoplast, partly by diffusion through aquaporins [133]. The apoplast, by contrast, often produces ROS intentionally via RBOHs during stress sensing and signalling. These temporary oxidative pulses are limited by apoplastic peroxidases and redox-active phenolics or flavonoids, which prevent excess ROS yet keep signal integrity [134,135,136]. Together, these compartment-specific antioxidant systems form a connected redox network: chloroplasts protect from light stress, mitochondria manage respiration, peroxisomes handle peroxide surges, the cytosol buffers ROS traffic, and the apoplast enables ROS-based signals [137,138]. This setup allows plants to balance their protective and signalling roles as conditions change.

## 5. Antioxidant-Mediated Tolerance to Specific Abiotic Stresses

Plants face a wide range of abiotic stresses, non-living environmental factors such as drought, salinity, and extreme temperatures, that disrupt cellular redox homeostasis, or the balance between antioxidants and reactive molecules in cells, by triggering excessive production of ROS [20,23]. ROS can cause cellular damage but also serve as crucial signalling molecules [1,38]. To counterbalance ROS overaccumulation, plants rely on sophisticated antioxidant systems. These systems mitigate oxidative stress, a harmful condition resulting from an imbalance between ROS and antioxidants, and facilitate acclimation [15,34]. Antioxidant defences operate through distinct yet often interconnected mechanisms. They are tailored to modulate ROS levels and maintain cellular equilibrium under diverse stress conditions (Table 1).

### 5.1. Drought Stress

Drought is the most severe abiotic stress affecting global agriculture. It causes yield losses of 40–70% in key cereals such as maize, wheat, and rice during severe events [139,140]. Water deficit induces stomatal closure, reducing CO_2_ assimilation by 50–70%. This disrupts the balance between light absorption and carbon fixation [140]. The imbalance increases electron leakage from photosystems I and II. This electron leakage generates O_2_^−-^ via the Mehler reaction at rates of up to 2–3% of the total electron flux [141]. Mitochondrial electron transport is similarly over-reduced, releasing superoxide mainly from complexes I and III [142]. Concurrently, peroxisomes produce substantial H_2_O_2_ during intensified photorespiration. In drought-stressed mesophyll cells, concentrations can exceed 100 µM [143]. These ROS act as signalling molecules to trigger defence pathways but also serve as toxicants, causing lipid peroxidation, protein carbonylation, and DNA damage [170]. A central defence mechanism involves a robust network of antioxidant enzymes to detoxify ROS. For instance, SODs convert O_2_^−-^ to H_2_O_2_. This prevents formation of –OH [28]. In drought-stressed wheat, SOD activity increases 2.5-fold. This increase correlates with a 45% reduction in MDA, a lipid peroxidation marker [59]. Peroxisomal CAT detoxifies accumulated H_2_O_2_. This results in a threefold increase in activity in drought-treated maize [110]. Similarly, APXs in chloroplasts, mitochondria, and cytosol precisely regulate H_2_O_2_ at signalling levels [138]. *Arabidopsis apx1* mutants accumulate 60% more H_2_O_2_ under drought. They suffer earlier PSII photoinhibition and show a 20% survival decline. In contrast, APX-overexpressing lines maintain Fv/Fm ratios 25% higher, indicating protective effects [64,139]. The AsA–GSH cycle acts as a pivotal redox amplifier, buffering ROS fluxes [171]. Drought elevates the GSH/GSSG from ~4:1 to nearly 9:1 in soybean, sustaining APX activity [140,171]. Wheat exhibits a 3.8-fold increase in GR during prolonged drought. This change ensures efficient GSH recycling [59]. Metabolomic profiling in barley (*Hordeum vulgare* L.) reveals a 2.5-fold rise in ascorbate and a threefold increase in GSH pools. This confirms synergistic antioxidant engagement [97].

Non-enzymatic antioxidants, such as tocopherols, are also key regulators of drought stress in maize. They preserve thylakoid membrane integrity and enhance zeaxanthin up to tenfold via the xanthophyll cycle. This boosts non-photochemical quenching, allowing excess energy to dissipate [128,135,170]. Compatible solutes–proline, glycine betaine, and soluble sugars–are accumulated. These solutes modulate osmotic adjustment by maintaining turgor and stabilizing cellular structures [19,27]. In maize during drought, proline rises from ~3 to 15 µmol g^−1^ fresh weight. This increase is accompanied by a twofold increase in APX and a 35% decrease in ROS leakage [171]. Beyond osmoprotection, proline directly quenches singlet oxygen. It also upregulates antioxidant genes through redox-sensitive transcription factors [172]. Glycine betaine stabilizes Rubisco activase and thylakoid membranes, thereby indirectly mitigating ROS production in photosynthesis [173]. Drought elicits ABA-mediated ROS bursts in guard cells via RBOHs at the signaling hub [62]. ABA induces apoplastic H_2_O_2_ accumulation (10-20 µM) within minutes. This activates Ca^2+^-dependent kinases and promotes stomatal closure [174]. Antioxidants buffer these transient ROS elevations, preventing oxidative damage and preserving signal fidelity. Systems-level analyses identify APX2, 2-Cys peroxiredoxins, and GR as core nodes integrating ROS detoxification with ABA- and DREB-driven transcriptional programmes [175]. Proteomic studies reveal that drought induces reversible cysteine oxidation in over 80 proteins, including Calvin–Benson and glycolytic enzymes. These proteins are subsequently reactivated through thioredoxin-dependent reduction [176]. These redox switches exemplify the intertwined regulation of metabolism and stress signaling. Overall, drought-responsive antioxidant systems do more than detoxify. They orchestrate a nuanced balance between signalling and oxidative defence. By safeguarding the photosynthetic apparatus, stabilizing membranes, coordinating osmotic balance, and modulating hormone crosstalk, antioxidants create a biochemical and molecular framework. This framework underpins crop resilience under water-limiting conditions.

### 5.2. Salinity Stress

Soil salinization affects approximately 20% of irrigated croplands and is also increasing in areas of rainfed agriculture, a problem that is expected to worsen with climate change [54,177]. High sodium (Na^+^) and chloride (Cl^−^) concentrations pose two primary challenges for plants: osmotic stress, which limits water uptake and mimics drought, and ionic toxicity, where Na^+^ and Cl^−^ displace essential nutrients such as potassium, calcium, and magnesium [100,144]. This disrupts ion balance, damages membranes, impairs enzyme activity, and reduces photosynthetic efficiency [145]. Mechanistically, salinity stress limits CO_2_ assimilation and increases photorespiration, leading to excess excitation energy directed toward oxygen, producing O_2_-^−^ in chloroplasts [146]. Additionally, Na^+^ influx depolarises membranes which triggers potassium efflux and activates RBOHs, leading to localized ROS surges that damage lipids, proteins, and DNA [147,148,149,150,151]. For example, rice seedlings exposed to 150 mM NaCl show a ~60% increase in MDA and a ~40% decrease in chlorophyll content, indicating membrane damage and a decline in photosynthetic activity [145].

In salt-tolerant barley, SOD and APX activities increase up to 2.3-fold and 1.9-fold, respectively, reducing H_2_O_2_ accumulation by 45% compared to sensitive genotypes [154]. In rice, APX overexpression improves seedling survival under salinity by 35%, stabilizing membranes and limiting ROS [178]. The glutathione system maintains redox homeostasis, as GR and GPX regulate the GSH/GSSG ratio, which decreases from ~12:1 to 3–4:1 during salt stress [152,153]. Arabidopsis overexpressing GR sustains higher ratios (~6:1), delaying senescence and preserving photosystem II efficiency [151,152]. Non-enzymatic antioxidants, such as ascorbate and glutathione, increase 2–3-fold in tolerant genotypes driving the ascorbate–glutathione cycle and recycling antioxidant capacity [179,180]. Ion and redox signalling cross-talk is essential in salinity acclimation [146]. Na^+^ influx disrupts K^+^ homeostasis and fuels ROS production, creating a feedback loop that modulates ROS waves, regulating ion transporters (e.g., *SOS1*, *NHX*) and osmoprotectant synthesis, such as proline and glycine betaine [181,182]. Application of ascorbate in salt-stressed wheat has been shown to improve K^+^/Na^+^ balance, lowering MDA by 35%, and raise relative water content by ~20%, enhancing metabolic function and osmotic adjustment [183]. Overall, the synchronized induction of antioxidant enzymes and metabolites delineates the threshold between salt-induced damage and adequate acclimation.

Soil salinization affects around 20% of irrigated croplands. It is rising in rainfed areas, a problem expected to worsen with climate change [54,177]. High sodium (Na^+^) and chloride (Cl^−^) concentrations pose two main challenges for plants: osmotic stress and ionic toxicity. Osmotic stress limits water uptake and mimics drought. Ionic toxicity occurs when Na^+^ and Cl^−^ displace potassium (K^+^), calcium (Ca^2+^), and magnesium (Mg^2+^) [100,144]. This disrupts ion balance, damages membranes, impairs enzyme activity, and reduces photosynthetic efficiency [145]. Salinity stress also limits CO_2_ assimilation and increases photorespiration. As a result, excess excitation energy is diverted toward oxygen, producing O_2_^-−^ in chloroplasts [146]. Additionally, Na^+^ influx depolarises membranes, triggers potassium efflux, and activates RBOHs. This process leads to localized ROS surges that damage lipids, proteins, and DNA [147,148,149,150,151]. For example, rice seedlings exposed to 150 mM NaCl show a ~60% increase in MDA and a ~40% decrease in chlorophyll content. These indicate membrane damage and reduced photosynthetic activity [145]. In salt-tolerant barley, SOD and APX activities increase up to 2.3-fold and 1.9-fold, respectively. As a result, this reduces H_2_O_2_ accumulation by 45% compared to sensitive genotypes [154]. In rice, APX overexpression improves seedling survival under salinity by 35%. It stabilizes membranes and limits ROS [178]. The glutathione system also maintains redox homeostasis. GR and GPX regulate the GSH/GSSG ratio, which drops from ~12:1 to 3–4:1 during salt stress [152,153]. Interestingly, *Arabidopsis* overexpressing GR maintains higher ratios (~6:1), which delays senescence and preserves photosystem II efficiency [151,152]. Non-enzymatic antioxidants, such as ascorbate and glutathione, increase 2–3-fold in tolerant genotypes. This drives the ascorbate–glutathione cycle, recycling antioxidant capacity [179]. Ion and redox signalling cross-talk is essential in salinity acclimation [146]. Na^+^ influx disrupts K^+^ homeostasis and fuels ROS production, creating a feedback loop. This modulates ROS waves and regulates ion transporters (e.g., SOS1, NHX). It also drives the synthesis of osmoprotectants, such as proline and glycine betaine [181,182]. Applying ascorbate to salt-stressed wheat improves K^+^/Na^+^ balance, lowers MDA by 35%, and raises relative water content by ~20%. These changes enhance metabolic function and osmotic adjustment [183]. Synchronized induction of antioxidant enzymes and metabolites marks the threshold between salt-induced damage and effective acclimation.

### 5.3. Heat Stress

Rising global temperatures pose a severe challenge to agricultural productivity by disrupting plant physiological, biochemical, and molecular functions [184]. Heat stress accelerates ROS production, leading to protein denaturation and destabilization of membranes and enzymes [26]. In chloroplasts, this excess thermal energy impairs the photosynthetic electron transport chain, increasing electron leakage to oxygen and generating O_2_^-−^ and H_2_O_2_. H_2_O_2_ levels can increase fivefold in heat-stressed leaves, while mitochondrial ROS output may double due to reduced respiratory efficiency [155,156,184,185]. Heat-induced increases in membrane lipid fluidity promote lipid peroxidation, with MDA levels rising 40–70% in wheat, maize, and rice, illustrating extensive oxidative damage [155,156,183,184,185].

To counteract these effects, plants activate coordinated antioxidant defences [186]. Enzymes such as SOD, APX, and CAT detoxify ROS in chloroplasts, mitochondria, and the cytosol to maintain photosystem integrity [187]. In rice seedlings at 42 °C, Cu/Zn-SOD activity rises 2.3-fold while APX and CAT increase two- to threefold, reducing MDA by half [188]. Overexpression of APX in tomato preserves Rubisco activase and photosynthetic electron transport, highlighting its critical role in heat tolerance [189]. Heat stress also unfolds and aggregates proteins, but antioxidants act synergistically with heat shock proteins (HSPs) to stabilize protein structure and maintain enzymatic function [155,156]. The AsA–GSH cycle buffers redox homeostasis, maintaining high GSH/GSSG ratios to support ATP synthase and Calvin–Benson cycle enzymes even under thermal stress [190]. Thioredoxins and peroxiredoxins reverse heat-induced thiol oxidation, as evidenced by *Arabidopsis* 2-Cys Prx mutants that exhibit elevated ROS and rapid photosystem II photoinhibition under heat stress [30,191]. Membrane preservation is critical, as lipid-phase antioxidants such as tocopherols and carotenoids quench singlet oxygen and lipid radicals [191,192,193]. Heat stress induces a two- to threefold increase in α-tocopherol in thylakoid membranes, preserving fluidity and stability [192]. Additionally, β-carotene and zeaxanthin generated via the xanthophyll cycle dissipate excess thermal energy, thereby protecting photosynthetic pigments [42]. *Arabidopsis* mutants deficient in α-tocopherol exhibit accelerated senescence and lipid peroxidation under heat stress, underscoring the protective role of α-tocopherol in plants [193].

Heat-induced ROS also act as signalling molecules activating heat shock transcription factors (HSFs), especially *HSFA2*, which induces both HSPs and antioxidant enzymes [194,195]. Hormonal crosstalk involving abscisic acid and ethylene further modulates osmotic regulation, stomatal closure, and water retention, thereby alleviating oxidative damage [196]. Transient H_2_O_2_ increases (~10 μM) directly upregulating SOD and APX, and establishing a feedback loop that primes antioxidant defences during and after heat episodes [197]. Field and multi-omics studies have consistently shown that a strong antioxidant capacity is correlated with heat tolerance and yield stability [198]. Wheat varieties with high CAT and APX activities suffer less than 15% yield loss under 38 °C compared to over 40% in sensitive types [157,199]. Heat-tolerant rice varieties accumulate up to 12 mM glutathione and exhibit 30% less electrolyte leakage, reinforcing the importance of redox balance in thermal resilience [188]. Overall, integrating enzymatic and non-enzymatic antioxidants with redox-regulated chaperones and signalling networks provides the basis for plant adaptation to rising temperatures, a trait crucial for food security under climate change.

### 5.4. Cold Stress

Low or chilling temperatures pose a severe metabolic challenge, especially for plants from warm climates. They disrupt membrane fluidity, impair enzyme function, and increase ROS levels, thereby exacerbating cellular damage [158]. Sensitive crops, such as rice, tomatoes, and maize, may suffer yield losses of up to 50–80% after cold snaps [158,159,160,161,200,201]. Cold reduces membrane fluidity by inducing a gel-phase state in lipid bilayers. This impairs ion transport and destabilizes the chloroplast and mitochondrial electron transport chains. The result is electron over-reduction and ROS surges [162,163]. For example, maize seedlings at 5 °C show a 3.5-fold increase in chloroplastic superoxide. Barley and tomato exhibit 40–70% rises in MDA, indicating lipid peroxidation and membrane injury [158]. Genotypes with higher unsaturation of membrane lipids, notably linolenic acid, maintain fluidity and reduce ROS leakage during cold stress [160].

Cold triggers the rapid induction of enzymatic and non-enzymatic antioxidants, such as SOD, which converts superoxide to H_2_O_2_ [162]. This is then detoxified by APX and CAT across cellular compartments [200]. In wheat and rice, SOD activity doubles within 24 h of chilling, whereas in barley, CAT activity increases from approximately 120 to 350 µmol H_2_O_2_ decomposed per minute per gram of protein, resulting in a 60% reduction in H_2_O_2_ levels [158]. Thylakoid-bound APX protects photosystem II by regulating H_2_O_2_ concentrations; *Arabidopsis* overexpressing tAPX retain 35% higher photosynthetic efficiency and 40% less lipid peroxidation under cold conditions [202,203,204]. The ascorbate–glutathione cycle balances redox status by regenerating antioxidants; in rapeseed at 4 °C, glutathione pools increase nearly threefold, raising the GSH/GSSG ratio from 3:1 to 8:1 and supporting APX activity [15,38]. Deficiencies in GR or DHAR lead to cold sensitivity, chlorosis, and ROS accumulation [23]. Cold acts as both a stress and a signal, with membrane rigidification activating calcium influx, triggering MAP kinase cascades, and activating the ICE1-CBF-COR transcriptional pathway [205]. H_2_O_2_ pulses (10–20 µM) activate CBF transcription factors, promoting the accumulation of osmoprotectants, such as proline and sugars, which stabilize membranes [158]. Exogenous ascorbate upregulates cold-responsive genes and reduces electrolyte leakage by 30%, improving cold tolerance [206]. However, excess ROS above 100 µM induces oxidative damage, disrupting thylakoid membranes and photosynthetic enzymes, underscoring the importance of redox homeostasis for cold acclimation [207].

Cold stress also disrupts mitochondrial function and carbon metabolism [200]. Mitochondrial alternative oxidase serves as an electron sink that limits ROS generation, while thioredoxins and peroxiredoxins maintain key enzymes in reduced, active states [28,207]. Cold-hardy varieties express up to 4 times more thioredoxins, enabling more flexible metabolic regulation [160]. Lipid-soluble antioxidants like tocopherols and carotenoids protect membranes by quenching singlet oxygen and lipid radicals; α-tocopherol increases two- to threefold within 48 h of chilling in Arabidopsis, reducing photobleaching and preserving photosynthesis [203,204]. In summary, antioxidant-mediated cold tolerance integrates membrane stabilization, rapid antioxidant deployment, and cross-talk between ROS and hormone signalling, enabling plants to mitigate oxidative bursts and maintain electron transport. This coordinated defence ensures survival in extreme cold and supports acclimation, which is critical for cultivation in temperate and high-altitude regions.

### 5.5. Heavy Metal Stress

Heavy metals (HMs), including cadmium (Cd), chromium (Cr), nickel (Ni), copper (Cu), and vanadium (V), pose a major abiotic threat. They disrupt metabolic processes and redox homeostasis, often driving excessive ROS formation (O_2_^•−^, H_2_O_2_, ^•^OH). This leads to oxidative damage to lipids, proteins, and nucleic acids [15,38,208]. Across diverse species, HM exposure commonly elevates H_2_O_2_ and lipid peroxidation (MDA), while reducing photosynthetic efficiency and growth. For example, Cd stress increases oxidative markers and impairs photosynthesis in Arabidopsis and cotton (*Gossypium hirsutum* L.). Cr and Ni can also compromise PSII performance and carbon assimilation [209,210,211,212]. To counter ROS surges, plants activate a coordinated antioxidant network. SOD converts O_2_^•−^ to H_2_O_2_ and CAT or APX remove peroxide, supported by GR-driven recycling within the AsA–GSH cycle [15,28,208,211]. Enhanced APX and GR activity can stabilize chloroplast redox status and improve tolerance under Cd or Cu stress. This is consistent with the requirement for sustained antioxidant turnover—rather than single-enzyme changes [213]. Non-enzymatic antioxidants (Asc, GSH, tocopherols, flavonoids, phenolics) also buffer ROS and limit chain reactions. GSH plays a dual role as a substrate for phytochelatin synthesis, linking redox buffering to its capacity for metal detoxification [214,215]. A key feature of heavy metal tolerance is chelation and sequestration, which reduces free cytosolic metal ions and limits Fenton-type ROS amplification. Glutathione-derived phytochelatins bind metal ions to form HM–PC complexes. These complexes are transported into vacuoles via ABC transporters, lowering reactive metal availability in sensitive compartments [209,216,217]. Metallothioneins (MTs), rich in cysteine residues, provide binding capacity for metals such as Cd, Cu, and Zn and contribute to ionic homeostasis [218]. Increased PC production under Cd stress reduces free Cd^2+^ and partially recovers photosynthetic function, for example, in Brassica juncea L. Coordinated induction of MTs and GSH-dependent enzymes is linked with improved tolerance to Ni and Cr in crop systems [205,219,220]. Hormonal and redox signalling networks also shape these antioxidant and chelation responses. SA, JA, NO, and melatonin modulate antioxidant gene expression and redox capacity. Exogenous treatments (such as melatonin, SA, or silicon) have been reported to enhance activities of core antioxidant enzymes and associated protective pathways under metal stress. These treatments often reduce oxidative injury and support photosynthetic performance [205,207,221,222,223,224]. ROS–NO interactions, including redox enzyme modifications such as S-nitrosylation, can affect enzyme stability and activity. This further reinforces plant defence outputs [220,222]. In summary, heavy-metal tolerance is an integration of several defences. Antioxidant systems buffer ROS, chelators restrict metal reactivity and compartmentalize ions, and signalling networks coordinate these defences across tissues and organelles. Together, these systems enable plants to maintain metabolism and growth in contaminated environments [215,220,222].

## 6. Genetic and Biotechnological Levers That Tune Antioxidant Network Balance

**Figure 3:**
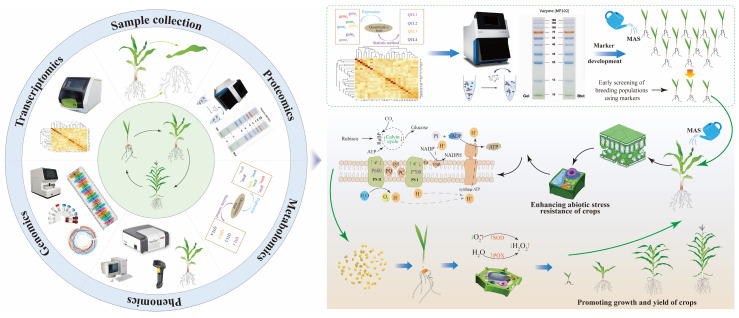
Integrative pipeline connecting stress-induced redox perturbation to deployable tolerance via omics, breeding, and antioxidant interventions. Left: Researchers collect samples from different developmental stages and contrasting genotypes under normal and abiotic-stress conditions. This enables multi-layer profiling using genomics, transcriptomics, proteomics, metabolomics, and phenomics. These approaches help discover candidate loci and biomarkers linked to redox control and stress tolerance. Right (top): Identified loci are translated into marker development. These markers are deployed through marker-assisted selection (MAS) for early screening of breeding populations. Right (middle): Omics-guided targeting of photosynthetic electron transport and related redox pathways links mechanistic antioxidant capacity to improved stress resilience. Right (bottom): Antioxidant-based interventions, such as priming or exogenous antioxidants, modulate the ROS and antioxidant balance. These changes are visualized as improved growth and yield stability under stress. Green arrows (↑/↓) indicate enhancement or inhibition after adding antioxidants. Red indicators (↑/↓) mark increases or decreases in response to abiotic stress relative to the normal state. Abbreviations: MAS, marker-assisted selection; QTL, quantitative trait locus; ROS, reactive oxygen species; SOD, superoxide dismutase; POX, peroxidase.

Section 3, Section 4 and Section 5 show that abiotic stress (environmental factors such as drought or extreme temperatures) disrupts the balance of ROS, and the effect depends on the cell compartment (specific locations within the cell, such as the chloroplast or mitochondrion). Under normal conditions, ROS are molecules that work in controlled signalling. But stress can lead to harmful ROS buildup if antioxidant defences (cellular systems that neutralize ROS) are overwhelmed. ROS metabolism and defence often overlap and support each other, so a single-gene fix rarely improves stress tolerance [225,226,227]. Section 6 outlines genetic and biotechnological ways to adjust antioxidant networks: (i) limiting ROS production by managing relevant enzymes and pathways, (ii) boosting detoxification by improving key enzymes (superoxide dismutase, ascorbate peroxidase/peroxiredoxin, catalase: SOD–APX/Prx–CAT), (iii) improving antioxidant recycling through the ascorbate–glutathione (AsA–GSH) and Trx-dependent systems, and (iv) targeting specific cell compartments and maintaining ROS signalling. These strategies are checked against stress responses and, where possible, crop yield data (Table 2). Traditional breeding has made some progress, but newer tools such as omics (comprehensive studies of cell molecules), transgenics (inserted genes), and genome editing (precise genetic changes) enable more targeted improvements across the whole network [Figure 3].

### 6.1. Transgenic Overexpression of Antioxidant Genes

Transgenic overexpression of antioxidant enzymes has been widely used to enhance tolerance to abiotic stresses by limiting ROS-driven damage to lipids, proteins, and DNA [258]. However, the effectiveness of this approach depends strongly on the gene selected, its subcellular targeting, and the specific stress context, given that ROS production is spatially and temporally structured [259,260,261]. Among these enzymes, SODs provide an upstream defence by converting O_2_^•−^ to H_2_O_2_, thereby reducing radical pressure at photosynthetic and respiratory electron transport sites [226,262]. Reflecting this, chloroplast-targeted Cu/Zn-SOD overexpression in tobacco improved photosynthetic performance and reduced photoinhibition under high light, while mitochondrial Mn-SOD in rice improved salinity tolerance by limiting respiratory ROS leakage and supporting energy metabolism [184,261]. SOD overexpression has also been associated with improved drought-related performance and symbiotic traits in legumes, underscoring its value as a buffer at ROS-generating “hotspots” [263]. Because SOD increases H_2_O_2_ formation, downstream peroxide-scavenging capacity becomes particularly important. In this context, APX operates effectively at signalling-range peroxide concentrations and can improve stress tolerance when appropriately expressed and targeted [15]. For example, cytosolic APX overexpression in rice improved salt-stress survival with reduced H_2_O_2_ accumulation, and thylakoid-associated APX in *Arabidopsis* reduced lipid peroxidation under high light, supporting chloroplast protection [179]. Notably, co-overexpression of SOD and APX in tobacco can yield additive protection, illustrating the advantage of coordinating consecutive steps rather than enhancing a single enzyme in isolation [148,262]. Furthermore, catalase provides high-capacity H_2_O_2_ removal, particularly relevant in peroxisomes during photorespiration, and CAT overexpression has been linked to improved drought- or salinity-related performance in crops such as maize and cotton, including reduced membrane leakage and better chlorophyll retention [25,43,120,264].

In addition to the enzymes described above, glutathione-dependent enzymes reinforce whole-cell redox buffering by sustaining recycling throughput. For example, GR overexpression can maintain a more reduced glutathione pool (a higher GSH/GSSG ratio) under stress, supporting continued APX/GPX turnover and redox stability, whereas GPX overexpression can reduce lipid peroxidation (e.g., lower MDA) and protect membranes under salinity [147,265]. Taken together, these examples show that antioxidant engineering can improve stress performance when it increases system throughput (detoxification plus recycling) rather than creating an unbalanced bottleneck. Several mechanistic principles emerge across studies. First, compartment targeting matters: expression in chloroplasts or mitochondria often yields stronger benefits than cytosolic expression because ROS must be intercepted near their sites of origin. Second, balanced pathway engineering is essential: elevating SOD without matching APX/CAT capacity can increase H_2_O_2_ accumulation and shift stress responses toward toxicity rather than acclimation [266]. Third, constitutive overexpression can impose growth penalties under non-stress conditions due to metabolic costs and disruption of ROS-dependent developmental signalling, motivating the use of inducible promoters and stress-responsive designs [267]. Illustrative successes, such as improved cold-related performance in potato with Cu/Zn-SOD, enhanced drought-related root function in chickpea with APX, and delayed senescence/extended photosynthesis in maize with GR, support the potential of antioxidant transgenics when these design constraints are respected [268,269,270,271]. Overall, the field is moving toward multi-gene, compartment-targeted, and conditionally regulated strategies that coordinate ROS metabolism across compartments while preserving productivity.

### 6.2. CRISPR/Cas-Mediated Genome Editing to Enhance Antioxidant Capacity

CRISPR/Cas genome editing enables precise modification of endogenous antioxidant and redox-regulatory pathways. This approach offers a complementary route to classical transgenics for improving stress tolerance [228,229,230]. Edits can be introduced at native loci and, in many cases, without stable foreign DNA. CRISPR can adjust antioxidant network behaviour [231]. It helps reduce problems such as metabolic burden, mislocalization, and disruption of ROS-dependent signalling, which are associated with constitutive overexpression [232,233,234]. This precision is useful for engineering stress resilience while preserving yield. ROS detoxification must remain coordinated with development and signalling. CRISPR strategies for enhancing antioxidant activity generally fall into three categories. Knockout of negative regulators removes inhibitory constraints on ROS detoxification. For example, disrupting ROS-repressor genes in rice under salinity increased APX and SOD activity (≈1.8-fold), reduced lipid peroxidation (~35%), and improved seedling survival (~20%) [235,236]. In tomato, editing targets affecting ascorbate metabolism increased intracellular ascorbate pools and APX activity. This was accompanied by improved drought-related performance, including photosynthetic stability and fruit setting under water limitation [237,238,239]. These results suggest that relieving regulatory bottlenecks can shift redox balance toward controlled ROS buffering. However, benefits remain context- and target-dependent.

Cis-regulatory (promoter) editing provides a route to inducible or tissue-specific activation of antioxidant genes. This reduces the costs of continuous expression [240,241]. Coordinated upregulation of SOD/CAT/APX through promoter activation has been reported to reduce H_2_O_2_ accumulation under drought-like conditions [242]. Stress-responsive alleles remain largely silent under non-stress conditions and activate in response to salinity. This illustrates an attractive design principle: deploy detoxification capacity only when ROS input rises, minimising growth penalties [243,244,245]. Allele replacement or optimization can improve enzyme stability or kinetics under stress, such as heat-sensitive APX variants. This maintains detoxification when proteins are destabilized or when turnover becomes limiting [272,273]. A further frontier is regulatory rewiring by inserting hormone-responsive elements, such as ABA- or SA-responsive motifs. This more tightly couples antioxidant activation to endogenous stress signalling and preserves signalling fidelity [244,249]. Overall, CRISPR enables movement from “more antioxidants” toward network tuning. It removes bottlenecks, builds conditional control, and optimizes enzyme performance. These designs must consider system constraints such as NADPH supply, AsA/GSH recycling capacity, and compartment cross-talk [252,253,254,255,256,257].

### 6.3. Marker-Assisted Breeding for Antioxidant Traits

Although genome engineering has expanded experimental control over redox pathways, conventional breeding remains essential. This is especially true when regulatory constraints or market acceptance limit the adoption of engineered cultivars [228,231]. Marker-assisted selection (MAS) offers a practical way to exploit natural allelic variation in antioxidant enzymes and redox-buffering pathways. This approach improves stress resilience while operating within standard breeding frameworks [272,273]. QTL mapping and association studies have identified loci linked to antioxidant activities and metabolite pools. These discoveries enable breeders to track antioxidant-related traits without relying solely on slow or expensive phenotyping [274]. In rice, QTLs associated with increased SOD and APX activity under salinity have been reported. These explain a substantial fraction of phenotypic variation. Tolerant lines carrying these loci show stronger chloroplast ROS control and reduced membrane lipid peroxidation compared to susceptible backgrounds [275,276]. In wheat, drought-associated loci affecting GR activity and glutathione redox homeostasis have been mapped. Introgression of these loci has been linked to improved photosynthesis and grain filling under water deficit—traits that connect redox buffering directly to yield-relevant performance [277,278]. Similar patterns are reported in other crops. Lines enriched for antioxidant buffering capacity, such as CAT/GPX activity or higher ascorbate pools, often show improved chlorophyll retention (‘stay-green’) and better maintenance of productivity under drought or heat stress [279,280]. A consistent lesson is that antioxidant-associated effects are usually polygenic and network-based. Pyramiding multiple loci, for example, combining QTLs linked to both SOD and APX activity, can provide stronger reductions in oxidative damage markers and more robust biomass performance than single loci. This reinforces the idea that resilience derives from coordinated antioxidant capacity rather than from isolated enzymes [281,282]. Looking forward, MAS will be most powerful when integrated with genomic selection, high-throughput phenotyping, and multi-environment trials. This integration will enable the selection of redox-associated alleles that remain beneficial across variable field regimes [283].

### 6.4. Omics-Driven Approaches

A complete understanding of antioxidant function cannot rely on a single enzyme or metabolite. This is because ROS metabolism is dynamic, interconnected, and strongly context-dependent [284]. Omics technologies, including transcriptomics, proteomics, and metabolomics, now provide system-level views of redox regulation. These technologies offer increasing spatial, temporal, and quantitative resolution [285]. Transcriptome profiling (especially RNA-seq) consistently shows broad remodelling of antioxidant networks during abiotic stress. For example, salt-stressed soybean induces large sets of redox-related genes spanning SOD, APX, GST, and GR families. This induction often shows marked tissue specificity [286,287]. Single-cell transcriptomics further refines this picture by revealing cell-type specialization. For example, some cells show preferential induction of peroxidase/peroxiredoxin modules in guard cells, while others show glutathione/catalase-linked programs in mesophyll. This pattern is consistent with compartment- and function-specific ROS control strategies [288]. Time-course studies add a crucial kinetic dimension. These studies often show an early induction of core enzymatic detoxification (e.g., SOD/APX). A later activation of secondary metabolism and the production of protective compounds usually follows. These findings imply a staged deployment of antioxidant defences, rather than a uniform response [289,290].

Proteomics complements transcript data by capturing post-transcriptional and post-translational regulation, which directly controls enzyme activity and signalling [291]. Redox proteomics has identified many stress-responsive thiol-modified proteins. These include Calvin–Benson cycle enzymes, photosystem components, and regulatory kinases. This supports the view that reversible cysteine oxidation acts as a redox “switchboard.” Such a switchboard connects ROS dynamics to metabolic reprogramming [292,293]. Thioredoxin- and peroxiredoxin-based systems counterbalance these modifications. This process links detoxification capacity to regulation of photosynthesis and stress signalling [75]. Metabolomics directly quantifies the sizes of the antioxidant pool and its redox states. This provides stress-specific biochemical signatures [287]. Drought and related stresses commonly shift ascorbate and glutathione pools and their redox ratios. These changes determine the throughput of the AsA–GSH cycle. High light can strongly increase xanthophyll-cycle components such as zeaxanthin, which support NPQ and photoprotection [61,294,295]. Together, these datasets emphasize that stress tolerance depends not only on component abundance. It also relies on turnover, redox state, and coordination across pathways.

The main value of omics lies in integrating datasets. Multi-omics network analyses can identify regulatory hubs and limiting steps (“bottlenecks”) in ROS detoxification capacity and reductant recycling. This process prioritizes mechanistically grounded targets for engineering or breeding [286,287,288]. Integrated studies in Arabidopsis, for example, have highlighted nodes such as APX2 and 2-Cys peroxiredoxins. These nodes are central connectors between ROS buffering and heat tolerance [296]. Beyond mechanisms, omics-derived signatures (transcript modules and metabolite markers) are increasingly used as biomarkers. These markers stratify germplasm and predict stress performance. They support translation into breeding pipelines, especially when combined with multi-environment validation [296]. In a translational context, omics-driven discovery complements intervention strategies. It provides the system-level constraints needed for rational design. Transgenics provide proof of concept that ROS damage can be reduced through antioxidant manipulation. CRISPR enables more precise tuning through promoter editing, allele optimization, and multiplex rewiring [228,229,230]. Marker-assisted and genomic approaches can then exploit natural diversity. These approaches deploy antioxidant-associated alleles into elite backgrounds across agroecological contexts [272]. Moving forward, the most robust route is likely an integrated framework. In this framework, omics identifies hubs and trade-offs. Engineering provides targeted perturbations. Breeding, plus field phenotyping, validates whether the altered redox regulation delivers consistent benefits to yield and stability under realistic, often combined-stress conditions [297,298,299,300,301].

## 7. Field-Facing Antioxidant Interventions Linked to Redox Mechanisms (Evidence and Limits)

This section focuses on field-facing interventions, including foliar antioxidant application, soil amendments that influence redox-active compounds, and priming treatments that modulate redox homeostasis during abiotic stress. We build on the compartment-resolved framework from Section 3, Section 4 and Section 5 and the genetic or biotechnological approaches outlined in Section 6. We discuss antioxidant-related practices only when there is a plausible or demonstrated mechanistic link to ROS control. We evaluate the evidence by considering limits that affect real-world performance, such as dose, timing, formulation or delivery, developmental stage, and genotype-by-environment interactions. Because manipulating antioxidants can preserve or suppress adaptive signalling, we also highlight situations in which exogenous inputs or priming strategies yield inconsistent or non-transferable results outside controlled environments.

### 7.1. Exogenous Antioxidant Application

The application of antioxidants directly is a fast and straightforward way to increase the redox buffering capacity of plants during exposure to abiotic stress [302]. These interventions act through two complementary mechanisms. First, they acutely increase the local antioxidant pool to quench ROS produced under stress insults before they damage cellular macromolecules. Second, they prime endogenous defense systems, thus prolonging protection beyond the treatment period [303,304]. Foliar sprays remain the most commonly applied method. They provide direct exposure to photosynthetic organs, where ROS production is highest [305,306]. Water-soluble antioxidants, such as Asc and GSH, penetrate leaf tissues through stomata and cuticular pores and scavenge H_2_O_2_, O_2_^•−^, and ^•^OH [55,62,63,64]. Ascorbate also functions as an electron donor for chloroplastic APX, reinforcing enzymatic detoxification [65]. Lipid-soluble antioxidants such as tocopherols stabilize thylakoid membranes and photosynthetic pigments. They also modulate apoplastic H_2_O_2_ signalling that coordinates stomatal closure and defence gene activation [116]. Data available confirm that there is a relevant physiological advantage when the right concentration and timing of formulation are used. For example, foliar spraying with 2 mM ascorbate in drought-stressed wheat increased photosynthetic rate by 25% and grain yield by 15% [55]. GSH sprays decreased H_2_O_2_ accumulation by 30% under Cd stress in rice [307]. Such applications are best before or at the time of early stress to achieve maximum acclimation benefit. This timing helps prevent an oxidative burst [308]. Effective foliar ascorbate concentrations generally range from 50–200 mg L^−1^, though millimolar levels are sometimes tested [96,97]. Over-application can induce osmotic stress or economic inefficiency. This emphasizes the need for calibration [115]. The absorption can be enhanced by using adjuvants. However, they must be used with caution due to their potential for phytotoxicity [174]. Seed priming is an economical approach to establishing antioxidant “memory” before germination. Pre-treatment of seeds with antioxidants or osmoprotectants induces a low-level oxidative signaling. This signaling up-regulates defense enzymes, such as SOD, APX, CAT, and GR, as well as the synthesis of osmolytes [309]. This metabolic priming accelerates germination and provides a lasting memory of protection against subsequent stress [310,311]. In maize, priming seeds with ascorbate or proline enhanced antioxidant enzyme activity by 1.5–3-fold in seedlings exposed to drought. It also increased vigor and establishment [312]. The antioxidant concentration and duration must be optimized for each crop species. Seed priming methods are readily transferable across different cropping systems [313]. Post-priming drying also needs to be well controlled to avoid contamination by microorganisms.

Application mediated by nanoparticles is an exciting frontier in sustained antioxidant protection [314]. Modifying SOD and CAT mimetics with engineered nanomaterials, such as cerium oxide nanoparticles (CeO_2_-NPs), results in catalytic “nanozymes.” These nanozymes shuttle between Ce^3+^ and Ce^4+^, mimicking SOD and CAT activities [315]. This redox-cycling is sustained without exhaustion. It allows continued ROS detoxification and, consequently, long-term protection. A 50 mg L^−1^ concentration of CeO_2_-NPs in tomato and cotton reduced ~40% ROS leakage, maintained chlorophyll content, and increased fruit or biomass yield under salinity and drought conditions [316,317]. Nanoparticles offer additional benefits over conventional antioxidants. These include sustained release, increased stability, and tissue-targeting specificity through surface modification or carrier matrices [6,192]. Nevertheless, the use of nanomaterials requires careful evaluation of their environmental risks [167]. Their persistence in soil and effects on the soil microbial community, nutrient cycling, and non-target organisms are still poorly understood [315]. Effective but non-disruptive concentrations should be defined through long-term, crop-specific trials [32,192]. A comprehensive, locally specific strategy holds the greatest potential for field resistance [196]. Otherwise, foliar antioxidant sprays provide quick protection during acute stress, such as heat spikes or short-term water deficits [318]. Seed priming is a strategy to promote early vigor and stress tolerance. Nanoparticle formulations can maintain redox homeostasis over extended growth periods [319]. When combined, such strategies (for example, antioxidant priming and foliar application at key stages of plant development) typically result in additive or synergistic effects [320]. In the future, dose–response optimization in practical field conditions is essential to optimize both application procedures and cost-effectiveness [321]. Concomitant research on the long-term ecological impacts, especially those of nanomaterials, is necessary to balance efficacy with sustainability. When properly applied, exogenous antioxidant applications can connect mechanistic redox biology with the practical requirements of crop management. Such applications provide immediate tools to stabilize yields by minimizing oxidative stress in an increasingly unstable climate.

### 7.2. Plant Growth-Promoting Rhizobacteria (PGPR) and Microbial Elicitors

Soil microorganisms play a key role in regulating plant redox biology. Their influence on oxidative balance goes beyond nutrient cycling and hormone interactions [322]. Plant growth-promoting rhizobacteria (PGPR), such as *Pseudomonas fluorescens* L., *Bacillus subtilis* L., and *Azospirillum brasilense* L., prime the antioxidant defense system and boost abiotic stress tolerance [323]. These microbes colonize the rhizosphere and root cortex, where they form a biochemical interface that helps control primary metabolism and maintain redox homeostasis [324]. The stress relief from PGPR comes from redox-dependent signaling. PGPR release phytohormones (including IAA, cytokinins, and gibberellins) and volatile organic compounds that adjust stomatal conductance, stimulate osmolyte synthesis, and activate antioxidant genes [325]. Under conditions such as drought or salinity, their inoculation consistently increases antioxidant enzyme activity [326]. For example, *Pseudomonas putida* L. inoculated into maize increased APX and GR activities by about 2-fold and reduced MDA by 50%, thereby improving membrane stability [327]. *Bacillus subtilis* L. in salt-stressed wheat increased SOD and CAT activities by 80–120%, which helped maintain photosynthetic efficiency and Fv/Fm [328]. PGPR also boosts non-enzymatic antioxidants. In tomato and rice, *Pseudomonas fluorescens* L. inoculation doubled the ascorbate and glutathione pools, supporting the ascorbate–glutathione cycle and promoting the synthesis of tocopherols, phenolic acids, and flavonoids [329,330]. This wide-reaching metabolic reprogramming shows that microbial priming extends beyond enzyme activation and creates broad redox optimization [325].

Mechanistically, induced systemic tolerance (IST) originates from microbeplant signaling [331]. Recognition of microbial molecules, lipopolysaccharides, flagellin peptides, or siderophores, by plant pattern-recognition receptors (PRRs) triggers transient ROS bursts via RBOHs, activating MAPK cascades and transcription of antioxidant genes [331,332]. This early signalling establishes a preemptive defense state that accelerates detoxification when stress occurs. PGPR also reshapes root architecture and ion balance, indirectly alleviating oxidative stress [331]. In saline soils, *Bacillus amyloliquefaciens* L. improved the K^+^/Na^+^ ratio by 30% and doubled APX and GR activity, sustaining photosynthesis and biomass. Enhanced nitrogen assimilation and organic acid exudation further contribute to redox stability [333]. Beyond living bacteria, microbial elicitors such as chitosan, β-glucans, and fungal metabolites mimic microbe-associated molecular patterns (MAMPs) to activate defense signaling [334]. These compounds induce systemic resistance through controlled ROS bursts and hormone crosstalk involving jasmonic acid and ethylene [335,336]. For example, foliar chitosan (100 mg L^−1^) in salt-stressed soybean increased CAT and SOD activity by 75–90% and phenolic content by 2.5-fold, lowering H_2_O_2_ by ~40% [337]. Similarly, β-glucan treatment in rice enhanced APX and peroxidase activity, stabilized chlorophyll, and reduced ion leakage under drought [338]. Such elicitor-triggered oxidative bursts serve as priming cues that strengthen antioxidant responses without causing cellular damage [334,335,336].

Using PGPR and microbial elicitors provides an environmentally friendly approach to redox resilience [334]. These biological tools strengthen antioxidant networks, which reduce stress-induced ROS. This also protects photosynthetic and growth processes [328]. Their effects last through both vegetative and reproductive growth, unlike the short-term effects of chemical sprays [334]. Future research should identify specific redox signaling patterns in strains, develop compatible microbial mixtures for different crops and soils, and combine inoculation with molecular breeding and precision farming [338]. However, persistence in the field, strong colonization, and environmental changes remain major challenges [339]. Even with these challenges, microbial priming moves the approach from reactive protection to proactive redox management. It forms a science-based plan for sustainable stress resilience in crops.

### 7.3. Biofortification Strategies

Biofortification (plant nutritional quality improvement) is a sustainable approach that increases oxidative stress adaptation and enhances plant nutritive value [340]. Unlike transient antioxidant sprays, it creates heritable and stable modifications to redox-active metabolites through genetic engineering, genome editing, and conventional breeding [341]. High pools of ascorbate, glutathione, tocopherols, and carotenoids detoxify ROS during stress and act as metabolic buffers [116,155,193]. Ascorbate biofortification focuses on enhancing flux through the Smirnoff–Wheeler and D-galacturonate reductase (GalUR) pathways [193]. Overexpression of GalUR in tomato increased fruit ascorbate 2.5–3-fold, improving stress tolerance and post-harvest life [342]. Overexpression of GDP-L-galactose phosphorylase (GGP) in rice and lettuce enhanced ascorbate content by 60–150% and protected chlorophyll from salinity-induced destruction [343]. These enhancements are reflected in the AsA–GSH cycle, which requires continued NADPH turnover and H_2_O_2_ detoxification via APX [72,76]. Glutathione biofortification targets key enzymes of the cysteine and γ-glutamyl pathways [65]. Overexpressing γ-glutamylcysteine synthetase (GSH1) or GR in *Arabidopsis* and rice increased glutathione pools 2–3-fold, raising the GSH/GSSG ratio and reducing membrane lipid damage by 40–50% [340]. These plants showed better tolerance to Cd, ozone, and salinity, while larger glutathione pools also aided recycling of ascorbate and tocopherol [73]. Carotenoid pathway engineering is a typical example [124]. Golden Rice, which contains both the *psy* and *crtI* genes, accumulated 30–35 μg g^−1^ of endosperm β-carotene, a precursor of vitamin A and a strong scavenger of singlet oxygen [341]. Similar approaches in maize and cassava (*Manihot esculenta* L.) resulted in twofold to fourfold increases in carotenoid levels, enhancing stress tolerance and yield [125,341]. Overexpression of γ-TMT increased tocopherol levels by 35–45% in wheat and soybeans, reducing seed aging and improving membrane stability during drought and heat stress [342]. α-tocopherol inhibits lipid oxidation and, with ascorbate, stabilizes redox states within plastids [343].

Recent innovations in CRISPR/Cas technology make targeted, DNA-free fine-tuning of antioxidant pathways possible [246]. For example, editing the GGP and GalUR promoters in tomato and rice has led to 30–70% increases in ascorbate levels. This method does not require transgene insertion [344]. Additionally, regulating the expression of repressors such as *SlAPX4* or *VTC2* can also increase endogenous antioxidant capacity. These plants show improved heat and drought stress tolerance [345]. Transferring stress-responsive cis-elements into promoters of antioxidants allows inducible regulation. This strategy conserves metabolic resources during optimal conditions but enables rapid antioxidant production during stress [231]. In summary, biofortification creates a win-win outcome. It enhances plant stress tolerance and improves human nutrition by raising levels of vitamin C and E, β-carotene, and phenolics [335]. Fresh market tomato lines high in ascorbate show 25% greater yield stability under drought and 30% firmer post-harvest fruit. These findings suggest a link between improved antioxidant metabolism and better agronomic and nutritional performance [346,347]. Combining biofortification with microbial priming, exogenous application, and CRISPR-based regulation gives plants a multifaceted oxidative stress defense mechanism [246,346]. The challenge is to refine metabolic fluxes and remove growth–defense trade-offs. Customizing antioxidant increases to crop physiology and environment is needed. Advances in systems biology, metabolic modeling, and breeding technologies will support the development of climate-resilient, nutrient-rich crops. These crops can sustain productivity and human health under increasing environmental stress.

### 7.4. Translating Antioxidant Modulation into Yield and Crop Quality Under Field-Relevant Stress Regimes

Many studies quantify antioxidants using biochemical proxies such as ROS, MDA, enzyme activities, and electrolyte leakage. However, these markers often fail to predict harvest outcomes. To enable translation, antioxidant interventions must be directly linked to yield components. These include maintaining photosynthetic duration, optimizing assimilate partitioning, improving flowering and fruit set, enhancing grain filling, and increasing harvest index. Evaluation should also focus on quality traits, nutrient density, firmness, shelf life, and seed longevity, not just seedling survival under stress. The evidence summarized above demonstrates this linkage in selected systems. For instance, tomato lines with elevated ascorbate show more stable yields under drought and firmer post-harvest fruit. Antioxidant capacity tracks with heat-stress tolerance in cereals as well. However, robust deployment needs both benefits under stress and penalties under non-stress conditions to be reported. This is due to potential trade-offs between growth and defense. Performance testing across varied environments and combined or sequential stresses is necessary, since ROS regimes and resource constraints often interact non-additively. Future antioxidant engineering and management should make yield and quality primary endpoints. Use redox measurements to explain and predict these outcomes.

## 8. Challenges and Knowledge Gaps

Despite progress in identifying antioxidant components and stress phenotypes, translating findings from controlled studies into the field remains inconsistent. Plant redox regulation is dynamic and compartmentalized. It also includes feedback loops. Changing one part can trigger compensatory responses or alter ROS signalling. The key is not just boosting “antioxidant capacity” but adjusting the redox balance in a predictable way. This means keeping ROS at helpful levels while avoiding long-term oxidative damage if environments change. Measurement is the first challenge. ROS and redox states are highly localized and brief. For example, H_2_O_2_ acts for seconds to minutes over micrometre distances. Hydroxyl radicals react where they are formed. Without compartment-specific quantitative data, studies rely on endpoint markers to infer ROS patterns. This hinders comparisons across species, tissues, or stress intensities. While genetically encoded probes, redox reporters, and multi-omics approaches are advancing, high-resolution mapping of ROS and antioxidant turnover in intact tissues is still limited. This is especially true in the field.

Another barrier is network nonlinearity and coupling. Increasing one enzyme’s activity can change the ROS pattern, not just decrease stress. For example, boosting superoxide dismutase can raise H_2_O_2_ levels. If peroxidase and reductant recycling do not keep up, defense signals or senescence may change. There is also a metabolic and developmental cost. Sustained antioxidant increases draw on carbon, nitrogen, and reductants. Excessive ROS removal can suppress essential developmental or stress signals. These factors explain why constant overexpression can improve tolerance in labs but lower growth or yield under non-stress conditions. Regulating antioxidants with stress-responsive, real-time systems is more promising. Field conditions create extra complexity. Stresses rarely occur alone; drought may combine with heat, salinity with poor nutrients, and temperature swings with oxidative bursts. These combinations mean ROS and antioxidant demands are not additive. Single-stress experiments cannot predict these effects. A key gap is understanding how the redox balance is reset during combined or sequential stresses. It is still unclear which intervention points are helpful or harmful under these conditions. Closing this gap needs factorial or sequential stress tests and compartment-specific redox tracking. Yield and quality measures are needed across reproductive development.

Environmental differences and genotype-by-environment interactions still limit translation. Redox processes differ with genotype, stage, tissue, microclimate, and stress timing. When and where you sample can change outcomes. To address this, use integrated phenotyping pipelines. Methods include non-destructive imaging, remote sensing, and, when possible, in situ redox reporting. Validate in multiple environments and use predictive models. These models should link ROS flows, antioxidants, reductant supply, and growth costs. This lets you assess interventions as whole-system designs rather than just single-gene changes.

## 9. Future Perspectives

The next frontier in plant redox biology is to convert mechanistic insight into robust, yield-stable field interventions. This will require moving beyond static “more antioxidant” strategies. Future work should focus on dynamic control. Regulatory circuits should activate detoxification and recycling only when ROS exceed physiological thresholds. At the same time, signalling needed for acclimation and development must be preserved. Synthetic biology tools, stress-responsive promoters, tunable regulatory modules, and compartment-targeted designs provide practical ways to implement this control in crops. Genome engineering is also shifting away from coarse overexpression. The field now uses precision tuning, including multiplex CRISPR, base editing, and prime editing. These approaches generate allelic series that adjust enzyme kinetics, localization, and redox-sensitive residues without adding foreign DNA. Target selection must consider system constraints, NADPH supply, glutathione/ascorbate recycling, and organelle cross-talk. This helps avoid stress tolerance that reduces yield under normal conditions. Building predictive measurement and modelling infrastructure is equally important. Integrating transcriptomics, proteomics, metabolomics, and redox flux with digital phenotyping and data-driven models enables clear, testable design rules for shifting the redox rheostat under changing field conditions. Microbiome-based strategies offer a complementary, potentially low-input approach. They can stabilize redox buffering via hormone–ROS crosstalk and metabolite support. However, success depends on colonization stability, soil context, host genotype, and formulation methods. Translation ultimately requires integrated breeding pipelines. These must combine natural diversity, genomic prediction, targeted editing, and multi-environment validation. Only in this way can resilience traits deliver consistent benefits without hidden yield costs.

## 10. Conclusions

This review advances a unifying interpretation of antioxidant function in abiotic stress. Outcomes depend less on absolute antioxidant abundance than on compartment-resolved network architecture, operating under the constraints of reductant supply and recycling throughput. Framed as a quantitative redox rheostat, the same ROS species support acclimation when restricted to specific concentration–time windows; conversely, they cause injury if fluxes are sustained or misregulated in space. Thus, inconsistent results from antioxidant manipulation arise when interventions ignore kinetics, compartmentation, and system-level coupling. A practical path forward relies on measurement and models. Priority needs include quantitative, in vivo ROS and redox mapping, identifying recycling and NADPH bottlenecks across tissues and stress phases, and mechanistically understanding inter-organelle coupling. Translational strategies are most likely to succeed with dynamic, conditional control, such as stress-responsive regulation, coordinated multi-node tuning, and genotype-by-environment validation, rather than with single-component overexpression. Following these principles, antioxidant biology can shift from reactive damage control to predictive design, enabling crop resilience strategies that work across real agricultural environments.

## Figures and Tables

**Table 1:** Enzymatic antioxidant responses contributing to oxidative stress mitigation in plants exposed to diverse environmental stresses.

Type of Stress	Plant Name	Experimental Conditions	Key Findings	References
Drought stress (20% PEG)	*Arabidopsis*	Controlled growth chamber conditions	*AgAPX1*, an ascorbate peroxidase gene from celery, is upregulated under drought stress and encodes a protein optimally active at pH 7.0 and 55 °C. Transgenic *Arabidopsis* overexpressing *AgAPX1* display increased *AsA* content (~1.4-fold), enhanced total antioxidant capacity (+21–29%), and improved drought tolerance.	[139]
Drought stress (15% PEG)	Soybean	Hydroponic experiment	SOD activity increased notably under severe drought by about 15%, while CAT and APX contents were elevated during drought stress, reflecting enhanced enzymatic antioxidant defense during water limitation	[140]
Drought stress (20% PEG)	Rapeseed	Hydroponic experiment	MDHAR and DHAR activities decreased under drought stress, while GSH levels increased by 31–26%, respectively. APX and AsA contents rose at lower stress levels, with GR remaining stable initially but increasing by 30% at drought stress.	[141]
Drought stress (20% PEG)	Rapeseed	Hydroponic experiment	Drought significantly increased oxidative stress (MDA + 82%, H_2_O_2_ + 131%), while proline pretreatment lowered them by up to 34% and 29%. Drought also boosted antioxidant enzymes, but osmolytes modulated responses differently (e.g., proline: MDHAR +15%, GR −12%; glycine betaine and trehalose varied). Overall, treatments improved glutathione redox status (↑GSH, ↓GSSG by 35–47%) and strengthened methylglyoxal detoxification via higher glyoxalase activity (up to +53%).	[142]
Drought stress (30% field capacity)	Tampala	Controlled growth chamber conditions	Key antioxidant enzymes such as SOD, CAT, APX, and GR are upregulated, helping to detoxify harmful ROS like superoxide radicals and hydrogen peroxide. Non-enzymatic antioxidants, including ascorbic acid and glutathione, also accumulate to maintain redox balance	[143]
Salinity stress (99 mM NaCl)	Tomato	Controlled growth chamber conditions	*FeSOD* overexpression strengthened antioxidant defences in tomato roots, stabilizing ROS levels and maintaining ordered microtubule structure under NaCl stress.	[144]
Salinity stress (300 mM NaCl)	Sweet potato	Petri dishes experiment	Overexpression of the *swpa4* peroxidase gene and CAT in sweet potato enhanced class III peroxidase activity (3–13×), significantly improving oxidative stress tolerance under H_2_O_2_ and salinity conditions	[145]
Salinity stress (100 mM NaCl)	*Arabidopsis*	Controlled growth chamber conditions	Overexpression of *AtGPXL5* (*glutathione peroxidase-like 5*) enhanced salt tolerance in *Arabidopsis* by maintaining lower H_2_O_2_ and MDA levels, elevating GSH content, and sustaining a more negative redox potential (≈−251 mV).	[146]
Salinity stress (200 mM NaCl)	Mustard	Controlled growth chamber conditions	Overexpressing cytosolic APX (*AtApx1*) in mustard improved salt tolerance (200 mM NaCl) by boosting ROS scavenging: H_2_O_2_ and MDA dropped ~2.8- and ~2.5-fold, while APX and GPX activities rose ~1.9- and ~1.5-fold. This strengthened the ascorbate–glutathione cycle, maintained redox balance, and reduced oxidative damage.	[147]
Salinity stress (100 mM NaCl)	Tomato	Hydroponic experiment	*SlMDHAR* expression, activity, and S-nitrosylation increase under salt stress, boosting ROS detoxification and redox balance. Transgenic plants overexpressing *SlMDHAR* show up to 48% higher germination, 2.3-fold longer roots, reduced ROS, and elevated antioxidant activities (SOD, CAT, POD, DHAR, APX, GR), enhancing salt tolerance effectively.	[148]
Salinity stress (400 mM NaCl)	Sugar beet and *Arabidopsis*	Controlled growth chamber conditions	*BvM14-MDHAR* from sugar beet is upregulated under salt stress, enhancing ascorbic acid regeneration and maintaining redox homeostasis. Overexpression in *Arabidopsis* improves salt tolerance by increasing MDHAR and DHAR activities, chlorophyll content, root growth, and AsA/DHA ratio, while reducing membrane damage.	[149]
Salinity stress (150 mM NaCl)	Kiwifruit	Controlled growth chamber conditions	*Actinidia chinensis* cytosolic APXs (*AcAPX1/AcAPX2*) are salt-induced antioxidants; when overexpressed in Arabidopsis, they improve salinity tolerance by raising APX activity (+31–39%), lowering H_2_O_2_ (−23 to −37%), and increasing total ascorbate and glutathione to strengthen redox homeostasis.	[150]
Salinity stress (150 mM NaCl)	Broad beans (Hassawi-3 and ILB-4347)	Controlled growth chamber conditions	Oxidative stress indicators (H_2_O_2_, MDA, electrolyte leakage) rose 60–120%, but their accumulation was 25–30% lower in ILB-4347 than in Hassawi-3. Antioxidant activities—SOD, CAT, GR, and AsA—increased by 25–85%, with ILB-4347 exhibiting 1.5–2 times higher enzyme levels than Hassawi-3.	[151]
Salinity stress (100 mM NaCl)	Wheat (Suntop and Sunmate)	Hydroponic experiment	Under salinity, the sensitive cv. Sunmate showed the highest MDA (greater oxidative damage), whereas tolerant lines (Suntop, CM72) had 1.5–2.2× higher SOD, POD, CAT, APX, and GR with earlier leaf peaks, improving ROS scavenging and cutting lipid peroxidation ~40–50% to maintain redox stability.	[152]
Salinity stress (8.55 mM NaCl)	Tomato	Controlled growth chamber conditions	AsA levels decreased, along with reductions in both reduced and oxidized glutathione (GSH and GSSG), leading to a lowered GSH/GSSG ratio. Concurrently, APX and GR activities increased by 28% and 14%, respectively.	[153]
Salinity stress (200 mM NaCl)	Common bean	Controlled growth chamber conditions	Under salt stress, key antioxidant enzymes rise strongly in tolerant genotypes (CAT up to +400%, APX +600–700%, GR +60%, with higher SOD), reducing lipid peroxidation (MDA −44–56%). Non-enzymatic antioxidants also increase (ascorbate +26–33%, flavonoids +47–70%), strengthening overall defense.	[154]
Heat stress (40 °C temperature for 5 h)	*Arabidopsis*	Controlled growth chamber conditions	Overexpression of *BcAPX* genes from *Brassica campestris* in *Arabidopsis* enhanced heat tolerance through increased APX activity and reduced oxidative damage. Specific transgenic lines (e.g., *BcAPX1-3, 2-1, 3-5*) showed 2–4× higher APX activity and lower H_2_O_2_ and MDA levels at 40 °C, indicating improved ROS detoxification and heat resilience.	[155]
Heat stress (40 °C temperature for 48 h)	Mung bean	Controlled growth chamber conditions	MDHAR and DHAR enzyme activities decline under stress, whereas APX and GR activities increase, contributing effectively to ROS detoxification and enhancing stress tolerance.	[156]
Heat stress (42 °C temperature for 12 h)	Tomato	Controlled growth chamber conditions	Under heat stress, tomato seedlings showed oxidative injury with MDA and H_2_O_2_ increasing by 48% and 60%, respectively. Antioxidant enzymes acted as the main defence, where SOD, POD, CAT, and APX activities rose by 11%, 47%, 75%, and 26% compared with control plants.	[157]
Heat stress (45 °C temperature for 12 h)	Rice	Controlled growth chamber conditions	Under 45 °C heat stress, rice seedlings showed severe oxidative damage, with MDA and electrolyte leakage rising >60%. This was mitigated by increased SOD and POD activity (~1.4–1.6-fold), which reduced H_2_O_2_ and O_2_^−^ by 30–35%. The upregulation of *OsCATB*, *Fe-SOD*, and *OsAXP1* genes confirmed enhanced antioxidant defence.	[158]
Cold stress (4 °C temperature for 48 h)	Potato	Controlled growth chamber conditions	Overexpression of *StSOD1* in potato enhanced antioxidant defence under cold stress. SOD activity in overexpressing lines rose 1.38-fold versus non-transgenic plants, while RNAi lines showed reduced activity. Increased SOD correlated with lower MDA content, and elevated POD (~1.24-fold) and CAT (~1.37-fold) activities.	[159]
Cold stress (4 °C temperature for 48 h)	Hardy orange	Controlled growth chamber conditions	Overexpression of *CsPIF8* enhanced cold tolerance in orange and transgenic tomato by activating SOD-mediated antioxidant defence. *CsPIF8* bound the E-box of the *CsSOD* promoter, elevating SOD expression and activity and reducing ROS and MDA accumulation.	[160]
Cold stress (4 °C temperature for 192 h)	*Arabidopsis*	Controlled growth chamber conditions	Overexpression of *AtSOD* and *CmSOD* in *Arabidopsis thaliana* markedly enhanced cold tolerance through stronger antioxidant defence. Transgenic lines showed 2–3 fold higher SOD activity and reduced superoxide accumulation, leading to lower oxidative damage after 4 °C exposure. Cold-responsive genes *AtCBF2*, *AtRD29A*, and *AtRD29B* were upregulated, with *AtRD29A* also induced by ABA, suggesting ABA-linked regulation.	[161]
Cold stress (4 °C temperature for 24 h)	Hardy orange	Controlled growth chamber conditions	Overexpression of *PtrbHLH* in *orange* enhanced cold tolerance by improving ROS detoxification. Transgenic lines showed lower ROS, electrolyte leakage, and MDA levels, along with higher CAT, POD, and SOD activities.	[162]
Cold stress (5 °C temperature for 24 h)	Rice	Controlled growth chamber conditions	Overexpression of wheat CAT in rice enhances cold tolerance by efficiently detoxifying H_2_O_2_, resulting in markedly higher CAT activity—especially under cold stress—while SOD and APX remain unchanged, confirming strengthened antioxidant defence and improved stress resilience.	[163]
Heavy metal stress (100 µM Cd)	Tobacco	Controlled growth chamber conditions	Overexpression of *BjCAT3* in tobacco improves Cd tolerance by enhancing CAT-driven ROS detoxification, leading to higher CAT activity, reduced H_2_O_2_ and MDA accumulation, and increased SOD and POD activities under Cd stress.	[164]
Heavy metal stress (200 µM Al)	*Arabidopsis*	Hydroponic experiment	Overexpression of *AtGR1* in *Arabidopsis thaliana* improved aluminum tolerance by boosting glutathione reductase activity, maintaining higher *GSH* levels and *GSH*/*GSSG* ratio, and reducing ROS and RCS accumulation.	[165]
Heavy metal stress (100 µM Pb)	Cotton	Controlled growth chamber conditions	Lead toxicity induces ROS that damage plant cellular components. In response, plants upregulate antioxidant enzymes (SOD, CAT, GPX, APX) to detoxify ROS, lowering oxidative damage indicators (MDA, H_2_O_2_, electrolyte leakage) and preserving cellular function under heavy-metal stress.	[166]
Heavy metal stress (300 ppm Pb)	Mustard	Controlled growth chamber conditions	Lead toxicity in plants triggers oxidative stress by increasing ROS, causing damage to cellular components. Antioxidant enzymes such as APX and GR are upregulated, increasing their activities by up to 57.84% and 51.97%, respectively, helping to regulate ROS and maintain redox balance. Elevated oxidative markers like H_2_O_2_ (up to 303.34%) and MDA (up to 34.82%) impair plant development, but enhanced antioxidant activity under heavy metal stress.	[167]
Heavy metal stress (600 ppm Cd)	Mustard	Controlled growth chamber conditions	Cd stress elevates H_2_O_2_ (up to 203%), MDA (41.3%), and electrolyte leakage (468%), indicating severe oxidative damage; antioxidant enzymes respond markedly, with CAT activity recovering and increasing up to 17.2% with treatment, GR rising up to 151%, and APX up to 192%, collectively protecting cellular function under Cd toxicity.	[168]
Heavy metal stress (1 mM Pb)	Wheat	Hydroponic experiment	Pb stress increased MDA (up to 179%) and H_2_O_2_ (95%) while suppressing antioxidant enzymes and ascorbate–GSH pools; GSH supplementation restored CAT and GPX, enhanced MDHAR (53%), DHAR (55%), GR (126%), SOD, and glyoxalase I/II (39% and 48%), reduced methylglyoxal (27%), improved leaf water content (13%), and lowered proline accumulation (24%).	[169]

**Table 2:** Genes used to improve abiotic stress tolerance in different crop plants.

Type of Stress	Crops	Techniques	Key Findings	References
Drought	Rice	CRISPR/Cas9	This study used eleven rice genes—*OsPDS*, *OsPMS3*, *OsEPSPS*, *OsDERF1*, *OsMSH1*, *OsMYB5*, *OsMYB1*, *OsROC5*, *OsSPP*, and *OsYSA*—to assess CRISPR/Cas9 editing efficiency and stress response. *OsDERF1*, *OsMYB1*, and *OsMYB5* regulate drought, salinity, and oxidative stress tolerance; *OsMSH1* maintains genomic stability; and *OsROC5* supports adaptive growth. *OsPDS*, *OsEPSPS*, and *OsYSA* acted as marker genes confirming successful editing.	[228]
Drought	Rice	CRISPR/Cas9	CRISPR/Cas9 knockout of *SRL1* and *SRL2* enhanced rice drought tolerance by boosting antioxidant defense. Mutants showed 45% higher survival, lower MDA, and 1.5–2× higher SOD and CAT activity than wild type. Proteomic data confirmed enrichment of antioxidant and stress-response proteins, indicating improved oxidative stress protection and drought resilience.	[229]
Drought	Rice	CRISPR/Cas9	*OsAAA-1* and *OsAAA-2* genes in rice negatively regulate drought tolerance; their overexpression reduces tolerance, while CRISPR-Cas9 knockout or RNAi silencing significantly enhances drought tolerance and grain yield without adverse growth effects.	[230]
Drought	Rice	CRISPR/Cas9	CRISPR/Cas9-induced mutations in *OsERA1* enhance drought tolerance through accelerated stomatal closure and increased ABA sensitivity without affecting leaf growth. *OsERA1* acts as a key negative regulator in ABA-mediated drought response, indicating its potential as a target for genetic improvement of drought tolerance and antioxidant activity in rice	[231]
Drought	Rice	CRISPR/Cas9	*OsPYL9* is an ABA receptor in rice that regulates drought stress tolerance by enhancing ABA accumulation and antioxidant enzyme activities (SOD, POD, CAT), and reducing MDA levels. CRISPR/Cas9-edited *OsPYL9* mutants show altered protein expression involved in circadian rhythms and abiotic stress responses, including proteins like *GIGANTEA* and pseudo-response regulators, which are central to ABA-activated signaling and ROS detoxification.	[232]
Drought	Rice	CRISPR/Cas9	*OsNAC14* is a drought-inducible transcription factor highly expressed during meiosis and in response to drought, salinity, ABA, and low temperature. Its overexpression enhances drought tolerance by activating genes involved in stress response, defense, and metabolism, notably directly upregulating *OsRAD51A1*, a key DNA repair gene.	[233]
Drought	Rice	CRISPR/Cas9	*OsPUB67* interacts with E2 enzymes and E3 ligases such as *OsRZFP34* and *OsDIS1* to form complexes that modulate drought response. It regulates ABA-dependent stress genes and enhances antioxidant activity, proline accumulation, and membrane stability during drought stress.	[234]
Drought	Rice	TALEN	*OsDERF1*, targeted by TALEN editing, is associated with enhanced drought resistance and plays a key role in regulating plant stress response pathways, including the modulation of antioxidant activity to improve abiotic stress tolerance.	[235]
Drought	Rice	ZFNs	A non-coding safe harbor locus on chromosome 3 was identified in rice using ZFN-mediated transformation, enabling stable integration of stress-regulatory genes such as *DREB*, *NAC*, *WRKY*, *SOD*, *CAT*, and *APX*.	[236]
Drought	Wheat	CRISPR/Cas9	Using CRISPR/Cas9, targeted editing of *TaDREB2* and *TaERF3* in wheat protoplasts confirmed their roles in drought and oxidative stress regulation. Both genes were efficiently and specifically mutated, and their upregulation under dehydration indicates key functions in enhancing stress tolerance and antioxidant defense in wheat.	[237]
Drought	Maize	CRISPR/Cas9	Maize *ARGOS8* negatively regulates ethylene sensitivity. Its elevated expression via CRISPR-Cas9 enhances drought tolerance without affecting yield under normal conditions.	[238]
Drought	Tomato	CRISPR/Cas9	*AITR* transcription factors (*AITR1*, *AITR2*, *AITR3*, *AITR4*, *AITR5*, and *AITR6*) negatively regulate ABA responses. CRISPR/Cas9 knockout mutants (*aitr256*, *aitr1256*, *aitr23456*, and *aitr123456*) show reduced ABA sensitivity and enhanced drought tolerance without growth or pathogen resistance trade-offs. Altered expression of ABA signaling genes *PYL4*, *PYL5*, *PYL6*, *HAI1*, and *ABF3* underlies improved stress resilience.	[239]
Salinity	Rice	CRISPR/Cas9	*OsbHLH024* acts as a negative regulator of salt tolerance. It reduces oxidative damage, indicated by lower ROS and MDA levels, and promotes balanced antioxidant enzyme activity. Molecularly, it upregulates *OsHKT1;3, OsHAK7*, and *OsSOS1,* enhancing Na+ exclusion and K+ retention to maintain redox and ionic balance, while downregulating *OsLEA3.*	[240]
Salinity	Rice	CRISPR/Cas9	CRISPR/Cas9-induced mutations in *OsRR22* enhance salinity resistance by modulating cytokinin signaling, which improves ion regulation and antioxidant defense. Mutant lines (*WPB106-cas-1* and *WPB106-cas-2*) maintain better redox balance and reduced oxidative damage under salt stress, showing that loss of *OsRR22* activates stress-responsive pathways for improved tolerance.	[241]
Salinity	Rice	CRISPR/Cas9	CRISPR/Cas9 mutants (*OsRR9* and *OsRR10*) showed improved tolerance, lower oxidative stress, and better Na^+^/K^+^ balance. Transcriptomic data revealed upregulation of ion transporters and antioxidant-related genes, indicating that *OsRR9* and *OsRR10* suppress stress-responsive pathways controlling ion homeostasis and redox balance under salinity.	[242]
Salinity	Rice	CRISPR/Cas9	The *OsNAC041* gene positively regulates salt stress tolerance in rice. Its expression increases under salinity, and CRISPR/Cas9 mutants lacking *OsNAC041* show higher ROS and MDA levels with reduced SOD, POD, and CAT activities, indicating impaired antioxidant defense. Transcriptome analysis revealed disrupted MAPK signaling, peroxisome function, and photosynthesis-related genes.	[243]
Salinity	Rice	CRISPR/Cas9	*OsSPL10* is a nuclear transcription factor that negatively regulates salt tolerance and positively controls trichome formation in rice. Knockout mutants show enhanced salt tolerance and glabrous leaves, while overexpression increases trichome density but reduces salt tolerance.	[244]
Salinity	Rice	S-adenosylmethionine decarboxylase	The *SAMDC* gene regulates stress and antioxidant activity by enhancing polyamine biosynthesis under stress conditions. Its ABA-inducible expression under NaCl stress increases spermidine and spermine levels, which act as antioxidants that stabilize cellular structures and scavenge reactive oxygen species, thereby protecting plants from oxidative stress.	[245]
Salinity	Barley	CRISPR/Cas9	The *HvITPK1* gene, along with other ITPK genes, regulates abiotic stress response and antioxidant defense via inositol phosphate metabolism. Its expression is induced by ABA and salt, and mutants exhibit altered stress sensitivity. The gene’s ABA-responsive promoter suggests it is transcriptionally regulated by oxidative and osmotic stress, positioning *HvITPK1* as a key regulator of stress signaling and antioxidant pathways.	[246]
Salinity	Barley	Overexpression by using 35S promoter	The gene *AVP1* enhances salinity stress tolerance by increasing shoot biomass and grain yield in transgenic barley under saline conditions without changing leaf sodium levels. *AVP1* promotes early growth and likely supports antioxidant activity by maintaining cellular ion balance and pH, helping plants manage oxidative stress during salinity.	[247]
Salinity	Soybean	CRISPR/Cas9	*GmAITR* genes in soybean are ABA-induced transcription repressors that regulate ABA signaling and stress responses. CRISPR/Cas9 mutants of *GmAITRs* show increased ABA sensitivity and enhanced salinity tolerance, with improved germination, growth, and yield under salt stress.	[248]
Salinity	Tomato	CRISPR/Cas9	The *SlHyPRP1* gene negatively regulates salt stress in tomato. CRISPR/Cas9-mediated precise deletion of its functional domains improved salinity tolerance by enhancing germination, growth, and survival under salt stress.	[249]
Cold stress	Rice	CRISPR/Cas9	CRISPR/Cas9 editing of *OsMYB30* enhanced cold stress tolerance and survival, while combined edits with *OsPIN5b* and *GS3* improved overall stress resilience. These gene edits help rice better manage abiotic stress, likely supporting antioxidant defences and improving tolerance to cold stress.	[250]
Cold stress	Rice	CRISPR/Cas9	The gene *OsAnn3* is crucial for rice cold tolerance, with increased expression under cold stress. Its knockout reduces survival and increases membrane damage, indicating a role in stress protection. *OsAnn3* likely helps stabilize membranes and supports antioxidant defences during cold stress.	[251]
Cold stress	Rice	CRISPR/Cas9	*OsPRP1* enhances cold tolerance in rice by regulating antioxidant enzymes and stress-related metabolites. Its knockout reduces survival, antioxidant activity, proline, chlorophyll, ABA, and ascorbic acid levels, leading to increased cold sensitivity.	[252]
Cold stress	*Arabidopsis*	CRISPR/Cas9	The *SPCP2* gene enhances plant stress tolerance by regulating senescence and antioxidant responses. Its expression under salt, drought, and hormonal stress supports cellular protection, helping maintain antioxidant balance and improve tolerance to abiotic stress.	[253]
Heat stress	Tomato	CRISPR/Cas9	The *SlAGL6* is a key regulator of heat tolerance, enabling fruit set under high-temperature stress. While not directly linked to antioxidant activity, future studies could investigate its role in stress resilience, potentially involving oxidative damage response.	[254]
Heat stress	Rice	Overexpression by using the Maize Ubi1 promoter	Overexpression of *Hsp101* enhances thermotolerance by maintaining protein homeostasis and protecting cells from heat-induced oxidative damage. Its activity supports the antioxidant defense network, reducing cellular stress and improving survival during severe heat stress.	[255]
Heat stress	Rice	Overexpression by using the Maize Ubi1 promoter	The *OsRab7* improves heat tolerance in rice by boosting antioxidant enzyme activities (CAT, SOD, APX, POD) and reducing oxidative damage markers (H_2_O_2_, MDA). It also upregulates key ROS-scavenging genes (*OsCATA, OsCATB, OsAPX2, OsSOD-Cu/Zn*) and other stress-responsive genes, enhancing osmotic adjustment and overall antioxidant defense under stress.	[256]
Heat stress	Rice	Promoter of Rca-a from *Oryza meridionalis*	The heat-stable *Rca* gene from *Oryza australiensis* maintains Rubisco function at high temperatures, preventing protein denaturation and indirectly reducing oxidative damage, thereby enhancing thermotolerance.	[257]

## Data Availability

No new data were created or analyzed in this study. Data sharing does not apply to this article.
